# Metal Laser-Based Powder Bed Fusion Process Development Using Optical Tomography

**DOI:** 10.3390/ma17071461

**Published:** 2024-03-22

**Authors:** Roy Björkstrand, Jan Akmal, Mika Salmi

**Affiliations:** 1 Department of Mechanical Engineering, School of Engineering, Aalto University, 02150 Espoo, Finland; jan.akmal@aalto.fi (J.A.); mika.salmi@aalto.fi (M.S.); 2 EOS Metal Materials, Electro Optical Systems Finland Oy, Lemminkäisenkatu 36, 20520 Turku, Finland

**Keywords:** additive manufacturing, 3D printing, stainless steel, process monitoring, parameter engineering

## Abstract

In this study, a set of 316 L stainless steel test specimens was additively manufactured by laser-based Powder Bed Fusion. The process parameters were varied for each specimen in terms of laser scan speed and laser power. The objective was to use a narrow band of parameters well inside the process window, demonstrating detailed parameter engineering for specialized additive manufacturing cases. The process variation was monitored using Optical Tomography to capture light emissions from the layer surfaces. Process emission values were stored in a statistical form. Micrographs were prepared and analyzed for defects using optical microscopy and image manipulation. The results of two data sources were compared to find correlations between lack of fusion, porosity, and layer-based energy emissions. A data comparison of Optical Tomography data and micrograph analyses shows that Optical Tomography can partially be used independently to develop new process parameters. The data show that the number of critical defects increases when the average Optical Tomography grey value passes a certain threshold. This finding can contribute to accelerating manufacturing parameter development and help meet the industrial need for agile component-specific parameter development.

## 1. Introduction

Metal laser-based Powder Bed Fusion (PBF-LB), often referred to as Selective Laser Melting (SLM), Direct Metal Laser Sintering (DMLS), or Direct Metal Printing (DMP), is an additive manufacturing (AM; i.e., 3D printing) technology for creating complex metal components without tooling by selectively fusing thin layers of powder with an energy source in the form of a laser or electron beam. Historically, the main uses of metal AM have been prototyping and tooling applications. Today it is also used for single or small series end-use component production [1], which is often combined with the optimization of design, for example, for low weight or maximum stiffness [2]. The technology and productivity of AM are evolving quickly, and the adoption of end-use components, including series production, is heavily increasing [3].

The main issues in the industrialization of technology are productivity [4] and quality assurance [5], along with the relatively high cost of production. Further, post-processing and total production processes are topics of discussion due to the high costs [6] and, to some extent, failed builds of components with difficult printability [7].

In order to push AM productivity further, larger and faster multi-laser equipment has been presented (e.g., SLM NXG XII 600™ or ePlus3D EP-M1550™) and the layer-by-layer process has been accelerated (e.g., Renishaw Tempus™). In material management, more flexible integrated material handling systems are being used (e.g., OneClick Metal, Mprint & Mpure cartridge system). Further, more straightforward post-processing and quality assurance are sought after through the use of, for example, support-minimizing designs [8], effective support removal technologies, and in-process quality monitoring systems [9]. Some recently introduced methods for improving productivity are laser spot size [10] and beam shape manipulation together with laser defocus and skin/core strategies [11]. Here, an increased laser spot size is used to melt the internal parts of a component more quickly, and beam shaping enhances melting in the fine details of a component.

However, at the core of each improvement are the process parameters that are deterministic for both productivity and quality [12]. It is also known that each individual machine configuration—of the tens of different models and the many different material suppliers available—can result in products of different qualities or face different printability issues. Even using the same material in different machines produces variance in mechanical properties, as was shown in cross-comparing research [13]. When comparing PBF-LB equipment and 316 L powders from two suppliers, the maximum difference in yield strength in the stress-relieved state was 7%. Moreover, Gong found remarkable variance in both PBF-LB and Electron Beam Melting (EBM) products manufactured with varying parameters [14].

The challenges of setting process parameters correctly are especially important in the production of components of optimized geometries. Complex shapes like triply periodic minimal surface gyroids [15] and lattices [16], where thin wall quality set challenges or unsupported downskins often form dross surfaces, lead to difficult printability using standard parameters. Due to the designed shape and possibly a reduced number of heat-conducting supports to enable internal cavities and low-angle downskins, for example, in the case of impellers [17], there can be process anomalies such as heat accumulation or overheating. The abovementioned issues were studied by Viale [18] and Druzgalski [19] among others. Printability difficulties can arise in the form of overly large deformations, failed features, or inadequate downskin surface quality [18].

There is reason to believe that component- and feature-specific parameter development is needed to reach uniform and adequate quality and to overcome the printability issues that originate from problematic components’ special features and design strategies [20]. Component-specific parameter development and engineering in AM can be compared to the “setup” process in traditional manufacturing; however, as opposed to traditional manufacturing setups that are required for every batch, when the component-specific parameter engineering is developed, it remains ready indefinitely for the idiosyncratic component.

**Figure 1:**
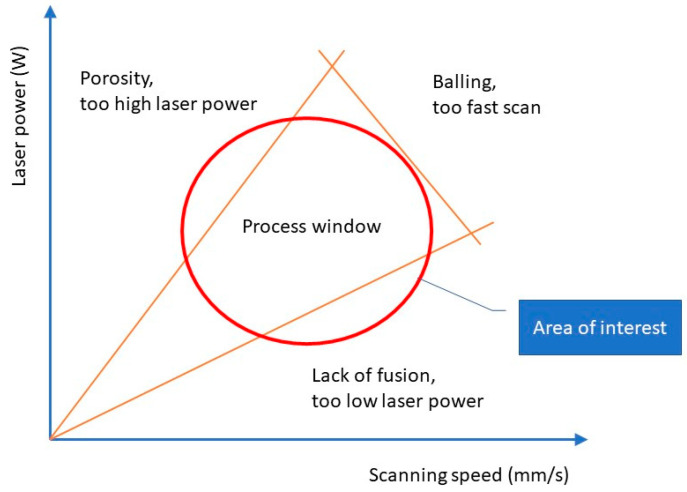
A process window for the Powder Bed Fusion process and the locations of typical defects on a laser power/scanning speed graph.

Earlier work on print quality concerning defects and process parameters has varied parameters from one extreme end of the spectrum to the other. This is because the intention has been to demonstrate the effects of different types of defects more clearly in the resulting parts. For example, du Plessis varied laser power from 80 to 350 W [12] and Schwerz varied power from 100 to 300 W and scan speed from 200 to 1600 mm/s [10]. These studies contributed to the concept of a process window (Figure 1) that reveals which kind of defect—porosity, lack of fusion, or balling—arises with certain combinations of parameters. Details of process window usage are described by SigmaLabs [21]. However, in an industrial setting, parameter engineering or optimization focuses on tuning parameters within the process window, not outside it.

Traditionally, in developing parameters for a new material, many samples are printed, tensile test specimens are made, and micrographs are prepared and analyzed to determine the best result for a given component. Such development work is often beyond the capabilities of the average service provider or manufacturing company operating a metal PBF-LB.

Research by Nafar [22], duPlessis [12], and Gong [14] showed that mechanical properties are directly linked to defects in the material. For fatigue, larger defects are located where crack initiation starts, and for tensile properties, a density less than 99% reduces the strength, and a density less than 95% does so significantly. Therefore, in this study, the focus is on defect characteristics affected by variance in process parameters that can be captured by Optical Tomography (OT).

Moreover, currently, it is typical that for each material, there are one to three standard parameter sets provided by the Original Equipment Manufacturer (OEM) focusing on either high quality or faster throughput. Usually, in OEM parameters, variables include layer height, laser power, contouring, or scan strategies. These standard parameters do not, however, consider part-specific needs that enable the reliable manufacturing of required challenging features. To some extent, operators can perform experience-based tuning to enhance manufacturing [23,24].

Therefore, in the industrialization of AM towards the series production of arbitrary components according to customer-specific quality and productivity needs, there is a growing need for process parameters that are not standard but, instead, component-specific [25]. The company Etteplan Oy reports a 25% build time savings from using customized process parameters [26]. There is also increasing business interest in developing other custom parameters, for example, to print amorphous crystal structures [27]. Research has also looked at controlling the microstructure via process parameter engineering in order to analyze and tailor materials’ mechanical performance [28,29]. This increases the need for parameter sets—or, more specifically, it requires an agile way of creating parameters to meet quality, performance, and productivity demands. Here, process monitoring systems offering an informative view of process thermal history can be of assistance.

Optical Tomography in AM was originally developed for quality assurance, in which validation of the print result is performed through the thermal history monitoring of consecutive builds [30]. The first build is quality-controlled, and after this, the identical thermal history of subsequent builds can be declared successful. Unlike the Optical Tomography traditionally used, for example, in the medical sciences, an OT apparatus in AM is constructed of a near-infrared camera located off-axis in the build chamber. This camera has a 5-megapixel detector providing approximately 0.1 mm resolution on a 250 mm × 250 mm platform. Parallelogram distortion due to off-axis location is corrected with optics so that the camera sees a square. The infrared camera captures several grayscale images (10 fps) during the fusing of a layer and filters the data to form a heat map showing the emitted radiation from the component through various color models. The radiation can be described as a unitless “grey value” (GV) that is proportional to the process temperature. Due to a narrow bandpass, typically around 900–940 nm, within the infrared range (750 nm–1000 µm), laser radiation (1064 nm) and plasma radiation (460–600 nm) are omitted. Based on empirical data and quality control activities, a correlation between hot and cold spots—called indications—and defects has been found [31]. OT software (EOSTATE Exposure OT, version 1.3.31.126) used in this study can automatically identify these indications offline based on user-defined algorithms. Besides image data, these systems also record numerical data and store it in a database in which each layer’s max, min, and mean GVs are included along with their standard deviations (STDs). These data can potentially be used in process parameter engineering.

The aim of this study is to find ways in which OT data can be used to fine-tune process parameters for ambiguous components. In this case, the parameters are well inside the process window with small differences. The hypothesis is that some defect-causing parameter combinations can be excluded, and Volumetric Energy Density (VED) suitable for the case can be chosen based on OT data. This is possible if the correlation between part defects and quality and OT raw data can be linked to parameter values or certain thresholds or guidelines regarding tolerable GVs. Finding this key link would support the establishment of parameter engineering.

## 2. Materials and Methods

### 2.1. Additive Manufacturing and Monitoring

**Figure 2:**
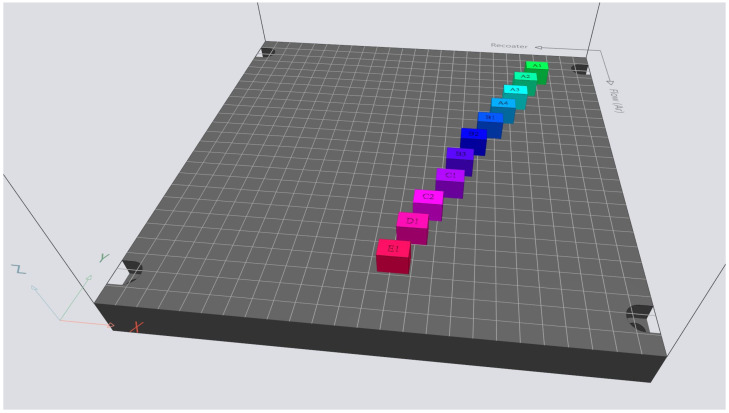
Layout of the specimens. The color coding indicates the parameter sets of the experiment.

In order to study the correlation between OT data, printing parameters, and the resulting print quality, 11 specimens (Figure 2) of 10 mm × 12 mm × 15 mm size were printed with varying scan speeds and laser power to obtain specific combinations of VED close to those normally used. An EOS M 290 (EOS GmbH, Krailling, Germany) machine with a 400 W Yb-fiber laser was used to print the specimens. The material was OEM non-virgin stainless steel 316 L powder (EOS GmbH, Krailling, Germany) printed with 40 µm layers. The powder size distribution (D_50_) was 37 µm. The other parameters were default values as follows: the rotating stripes scan strategy was 67°, the platform temperature was 80 °C, the hatch distance was 0.09 mm, and the shielding gas was argon (Argon N50, Woikoski Oy, Finland). The parameter variations in laser and scan speed are shown in Table 1.

The EOSTATE Exposure OT monitoring system (EOS GmbH, Krailling, Germany) was used to capture in situ process monitoring data. The OT system comprises an infrared camera with a bandpass of 900 nm at 25 nm full width at half maximum. This amounts to a spatial resolution of 125 µm/pixel on the build platform with a frame rate of 10 fps. Raw data images produced during the exposure (10 images × layer time in seconds) were filtered into a single image, where each pixel represents the maximum intensity of that layer [32]. OT data were exported in .csv file format from the process monitoring system, including GVs and statistics including max, min, and mean values, together with deviations per layer and per component. For this study, the OT provided 3000 lines of data. The data were processed statistically (calculations) and visually (data graphs) to find functional patterns and correlations.

### 2.2. Micrographs

**Figure 3:**
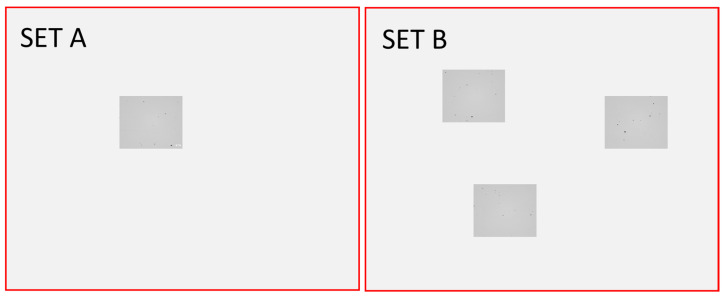
Microscope images covered only a subsection of the specimens (red square). On the **left**: illustration of a single image taken for Set A from a representative location. On the **right**: three random location images taken to produce more reliable data for set B. Grey areas are scaled to represent the actual size of the microscope images relative to the specimen.

The micrographs were analyzed via sample preparation. First, specimens were sanded with P800 and P1200 and then polished with diamond paste of 2000 and 3000 grit. Bright view optical microscopy (Zeiss Axiovert, Jena, Germany) was used to take images of the micrographs with a total magnification—optics and camera—of 3.15. Two data sets were formed. First, a representative image from each specimen was taken and used to validate image processing. This is referred to as set A. Then, to increase statistical reliability in capturing defects, three images from different random, non-overlapping locations of the specimens were taken—this is considered set B. A schematic of the microscopy is illustrated in Figure 3. The red squares represent the total area of the micrograph, and the darker grey areas represent images taken with the microscope—one image for set A and three images for set B to increase the coverage and reliability of the results. The image resolution was 2464 pixels × 2056 pixels (smaller squares in Figure 3), and one pixel corresponded to approximately 1.1 µm, which was adequate to reveal minuscule defects. One micrograph image corresponded to an area of 2.68 mm × 2.23 mm. The total area covered in set B was 17.9 mm^2^ per specimen, representing ~10% of the sample surface to improve statistical certainty.

The analyzed surfaces for sets A and B were in build direction (X-Z), showing melt pool defects such as lack of fusion and keyhole porosity. Afterward, the specimens were etched with Beraha II etchant, which is suggested by the company Steuers (Kopenhagen, Denmark) for austenitic stainless steels.

### 2.3. Data Analysis

Each image was processed using the software ImageJ version 1.54d (National Institute of Health, Bethesda, MD, USA and University of Wisconsin, Madison, WI, USA) to obtain numerical data. Bright view images were thresholded (auto) and binarized, after which the “analyze particles” function was used, which returned the number and size of the defects in pixels. Numerical values were stored in Excel (version 2308, build 16.0 Microsoft, Redmond, WA, USA) and joined with OT data, and different calculations were conducted to support the analysis.

**Figure 4:**
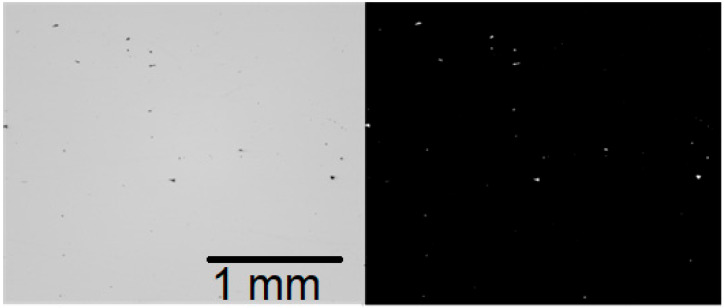
On the **left** is an example of a bright view image (micrograph A1). On the **right** is a binarized image that was used in the particle analysis. The image size was 2464 pixel × 2056 pixels or 2.68 mm × 2.23 mm.

For set A data, all particles were included, but for set B, a control parameter was set to return only defects larger than 10 pixels. This was to eliminate non-critical pores and noise from the calculations. A defect of pixel size 10 is inevitably smaller than 11 µm and thus clearly lower than the critical defect size in AM, which was identified as 20 µm by Prithivirajan and Sangid [33] through crystal plasticity simulations. Reijonen [34] took a similar approach to image analysis. Figure 4 is an example of a bright view image and its binarization, which was used to define areas of defect (white parts of the image).

The density was calculated using two methods as follows: (1) the processing of micrograph images using pixel values of the defect area compared to the total area and (2) the Archimedes principle (ASTM F3637-23 [35]). The histogram and list function of ImageJ software were used to obtain pixel data (number of black versus white pixels) for defect percentage calculation. In the image histogram, total pixel values for both solid (black) and defect (white) parts were listed in numeric form, so the numeric density could be calculated using Equation (1).

(1) $$N_{D}=\frac{T_{n}-W_{n}}{T_{n}}\;\times \;100$$

In the above equation, *N_D_* is the percentage of solid material in the sample, *W_n_* is the number of white pixels, and *T_n_* is the total number of pixels in the image. The Archimedes principle density test is performed first by weighing specimens directly on the scale and then weighing them submerged, utilizing a special rack with the scale. In the rack, a vat is supported outside the scale so that the scale only measures the weight of the specimen with affecting buoyancy force of the water in the vat. The density can then be defined by inputting these two weight values into Equation (2).

(2) $$N_{AP}=\frac{W_{a}}{W_{a}-W_{i}}\;$$

where *N_AP_* is the density, *W_a_* is the specimen weight directly on the scale, and *W_i_* is the weight of the specimen submerged. The density in the form of a percentage is achieved using a reference density as a divider. The literature reference value for 316 L density varies; here, the original equipment manufacturer declared a typical achieved value in AM to be 7.97 g/cm^3^. The scale used in the measurements was a Precisa 800M (PAG Oerlikon, Zürich, Switzerland) with a resolution of one milligram.

The data from both density sources was transferred to Excel (Microsoft, Redmond, WA, USA), connected, and analyzed to find statistical correlations and patterns. Excel’s CORREL function was used to test the interdependency of arrays of data. The CORREL function returns the correlation coefficient of two cell ranges. Correlation coefficients vary between 1 and −1, where the former indicates a strong positive correlation, and the latter indicates a strong negative correlation. In addition, ±0.4 is a considered the limit for moderate correlation, and 0 represents weak or no correlation. Correlations were further analyzed, and causalities were discussed. Patterns were observed both numerically and visually using graphs.

## 3. Results

### 3.1. Optical Tomography Grey Values

**Figure 5:**
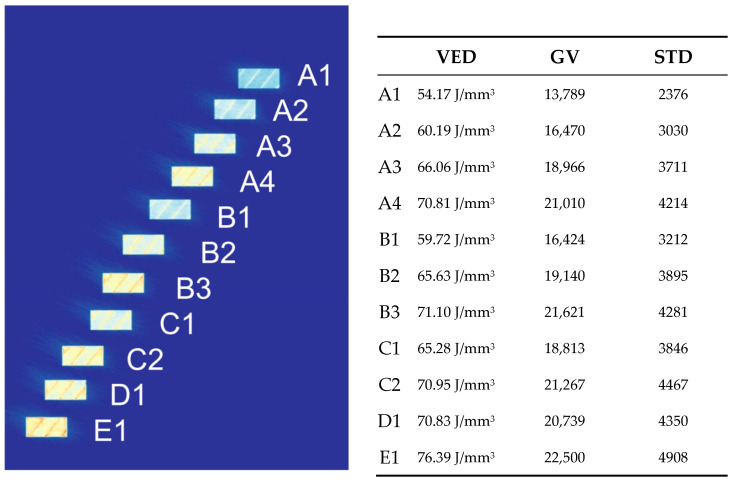
On the **left** is the Optical Tomography heat map image of the micrographs—colorized grey values span from 13,789 (A1) to 22,500 (E1). In the **right** table, the volumetric energy density (VED), grey values (GVs), and standard deviations (STDs) are provided.

First, VED and GVs were analyzed for correlation. There was a strong positive correlation of 0.99, which means that the average energy radiation received by OT was strongly in line with the set exposure values, defined by laser power and scan speed as the main parameters. Figure 5 presents an OT image and the corresponding VED and average GV values of the specimens, together with their standard deviations.

The GV correlation with the average defect size of sets A and B was analyzed, but it only had a moderate correlation (0.40, set A) or a weak correlation (0.17, set B). Set A also included small one-pixel defects, but for set B, defects under 10 pixels were excluded. The grey value correlation with density also showed similar results of a moderate or weak correlation (A = 0.49 and B = 0.35).

**Figure 6:**
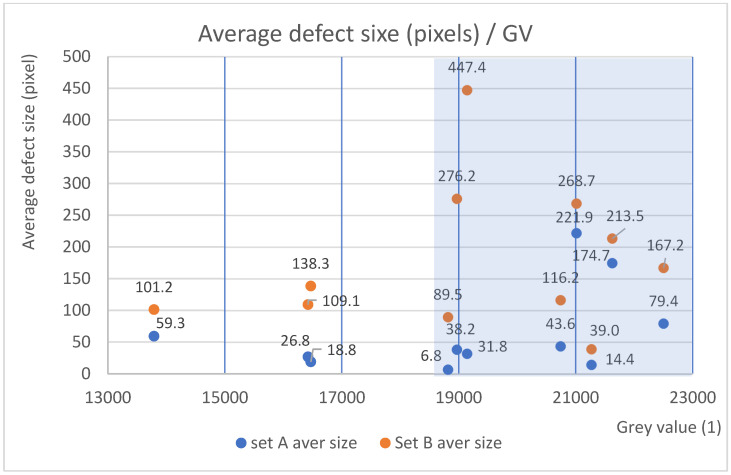
The average defect size (in pixels) increases when passing the threshold limit of 19,000 GV. The 230-pixel value approximately represents a spherical defect size of 17 µm. Each value on the chart represents one specimen’s average defect size.

Even though there was no clear correlation, patterns and trends were observed. Studying set A (the blue series in Figure 6) revealed that the average defect size in the specimens increases considerably when GVs exceed a value of 21,000 GV. For set B (the orange series in Figure 6), an increase in defect size was found to occur at GVs of 19,000 GV. Set B also had higher average defect size values in general, which is explained by the elimination of small defects below 10 px in image manipulation. A similar rise in defect size for sets A and B was observed also using the median defect size instead of the average.

**Figure 7:**
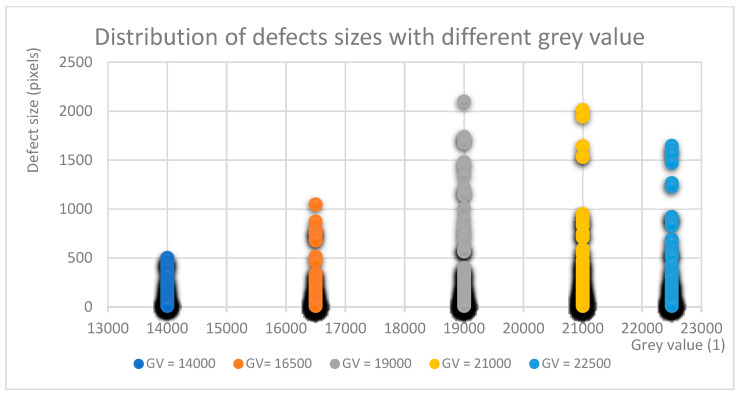
Distributions of defects at different grey value levels.

Another approach to the same data is shown in Figure 7, which shows the distribution of defects by size organized by GV. The three largest defects were excluded in order to enhance statistical reliability and avoid misleading scaling of the data. The distribution verified that large defects are more numerous when Optical Tomography results in grey values over 19,000 GV.

The first GV threshold (21,000 GV) corresponded to a VED value of 70 J/mm^3^, which was reached with the exposure parameters A4, B3, C2, D1, and E1. The lower GV threshold (19,000 GV) corresponded to a VED value of 66 J/mm^3^ and was reached with A3, B2, and C1.

Interestingly, the number of defects was low for these high VED parameters at the same time—that is, there were few but serious defects when using higher VEDs. The pattern of decreasing defect occurrence and increasing size can be seen in Table 2 for specimens that had equal laser power (A = 195 W, B = 215 W, C = 235 W) and for which VED was varied by scan speed. The only exception is the size of the errors in B2, which could have been due to some very large and random defects.

**Figure 8:**
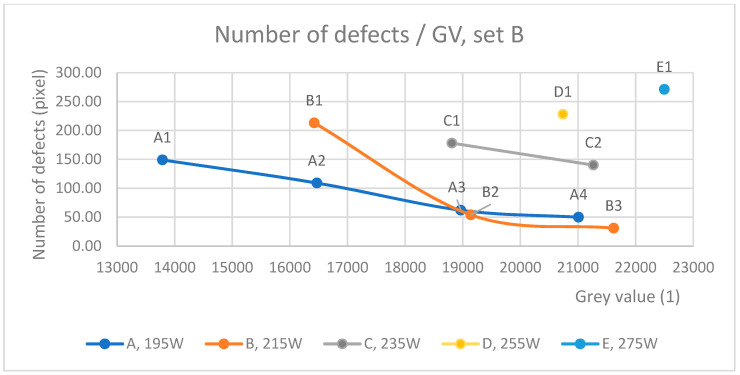
The number of defects by laser power. The number of defects decreases when grey values approach the threshold limit where large defects are formed. High laser energy was also correlated with the number of defects.

Previously, the defect size and distribution were analyzed. Additionally, the number of defects larger than 10 pixels was collected from the image analysis of set B, which is presented in Figure 8. Even though the number of defects found was at the higher end, the density was also relatively high, meaning that most of the numerous “defects” could be interpreted either as noise or as being so tiny that they were meaningless for structural strength.

A pattern existed for each laser power series, where the number of defects increased as laser power increased (from A to E) and fell slightly when scan speed slowed (from 1 to 4). Because sample B1 suffered from large random defects in one of the micrographs (Supplementary Materials Table S1), it was likely that specimens A2 and B1 would act similarly because their VEDs were equal. To this end, the number of defects increased after laser power increased over 235 W. For example, D1, with a laser power of 255 W, had triple the defects compared with equal VEDs in A4 and B3.

The scan speed for parameter group 1 was 1000 mm/s, for group 2, it was ~910 mm/s, for group 3, it was ~830 mm/s, and for group 4, it was 765 mm/s. All parameters with 1000 mm/s had more than 150 defects, while others had fewer. This does not necessarily mean stepping into the balling area of the process window, but it could possibly be a result of the longer melt pool track [21] causing minor voids.

By comparing similar VED (65 J/mm^3^) parameters A3/B2/C1, it can be deduced that the first two produced fewer defects than the higher-speed C1. Parameter C1 resulted in almost triple the number of defects. Visual and computational evaluation of the micrographs (see Supplementary Materials, Table S1), however, showed that A3 and B2 consisted of larger defects. The reason for this could have been that C1’s 235 W laser exposure, compared with A3’s 195 W and B2’s 215 W, was closing the upper limit of the conductive mode in the process window and entering a keyhole formation area.

The observation here is that the average GV does not recognize the number of defects. Most likely, the OT resolution was not accurate enough to capture the abnormal thermal behavior of all the smallest defects or thermal effects visible by the camera. The minimum number of defects, however, occurred when the GV was close to a threshold limit (19,000 GV or 21,000 GV, Figure 6). This happened with parameters A3 (195 W/820 mm/s) and B2 (215 W/910 mm/s). In addition, A4 and B3 produced similar numbers of defects, but according to Figure 6, they could be prone to larger defects due to high GVs. For example, for fatigue loads, large defects are unwanted. Also, high laser power parameters D1 and E1 produced a high number of defects, possibly indicating a process anomaly in the melt pool [36].

**Figure 9:**
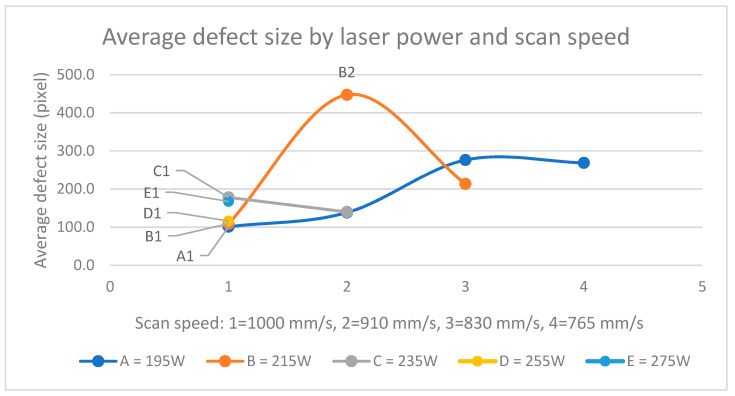
Average defect size (px) by laser power and scan speed.

The defect size was also observed similarly for a series of different laser powers (Figure 9). The laser powers in set B that shared the scan speed of 1000 mm/s (A1, B1, C1, D1, and E1) showed a lower defect size than the slower correspondents. The large defect in B2 distorted the visual observation, but all scan speeds below 830 mm/s had defects twice as large as those with 1000 mm/s speed. A1, B1, C2, and even D1 performed best here, even though the VEDs varied from A1’s 54 J/mm^3^ to D1’s 71 J/mm^3^. While the parameter B2 was possibly a random exception, the defect size tended to increase moderately when scan speed decreased. This could be an indication of nonoptimal melt pool dynamics [21], where a relatively low scan speed compared with laser power produces a wider melt pool and should be compensated with a greater hatch distance—the distance between subsequent laser lines—to avoid local overheating. The hatch distance was kept constant in this study.

### 3.2. Grey Value Deviation

**Figure 10:**
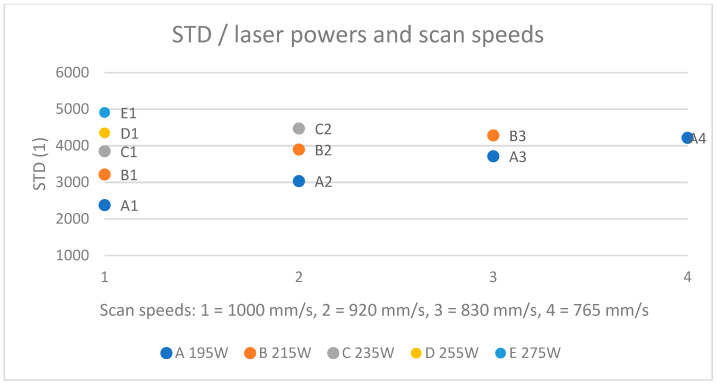
Standard deviation in OT data increases when laser power increases and scan speed decreases.

The earlier presented grey values and AM results (micrographs) were compared also with respect to grey value standard deviations (STDs). A high deviation in GVs between the layers could be an indication of an unstable process causing defects. Statistically, it seemed that the standard deviation was closely linked to the GV itself, being 17–21% of it for all specimens. If the STD is a marker of process instability, then the relative deviation (percentage of VED) should increase significantly more at the highest exposure values and be moderate at low values. It was found that the deviation increased as the laser power increased and also when the scan speed decreased (Figure 10); for example, A4 had double the deviation of A1. There was also a moderate negative correlation (set A −0.5, set B −0.35) between density and deviation but no correlation with the number or size of the defects. In the end, the deviation was not found to be indicative data for specimen geometries. However, for complex geometries or thin wall structures, the case may be different.

### 3.3. Optical Tomography Values and Process Window

**Figure 11:**
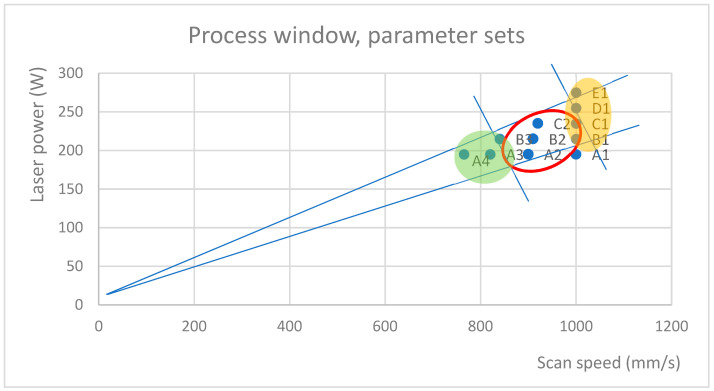
Process window showing the locations of the compared parameter sets. Suggested parameters are circled (red), and defect-prone areas are highlighted in green and yellow.

In Section 3.1, the interdependence of GVs, VEDs, and defects in the specimens was studied. This subsection joins these findings with specimen density and the process window. In Figure 11, the compared parameter sets are presented in the process window. Lines are drawn either to exclude either a high number of defects or a large average size of defects. The green area in Figure 11 represents the parameters that result in an increase in the number of defects due to a slower scan speed, while the yellow area is a high exposure area in which the average defect size was shown to increase.

The densities of the specimens were measured using two methods as follows: void areas of images and the Archimedes principle. Data from the Archimedes principle, which Spierings [37] suggested to be the most accurate method, was used to provide densities for this study, although the difference between the two methods was only 3%. An error analysis of the Archimedes principle with three repeated measurements showed an uncertainty of 2% for the results. The correlation between the density calculated from set B micrographs and the one achieved by the Archimedes principle was strong (value 1). For set A micrographs, the correlation was only moderate, which means that in set A, there is likely image noise in the form of <10 px defects affecting the result. Therefore, the use of the Archimedes principle and the prioritization of set B are justified.

**Figure 12:**
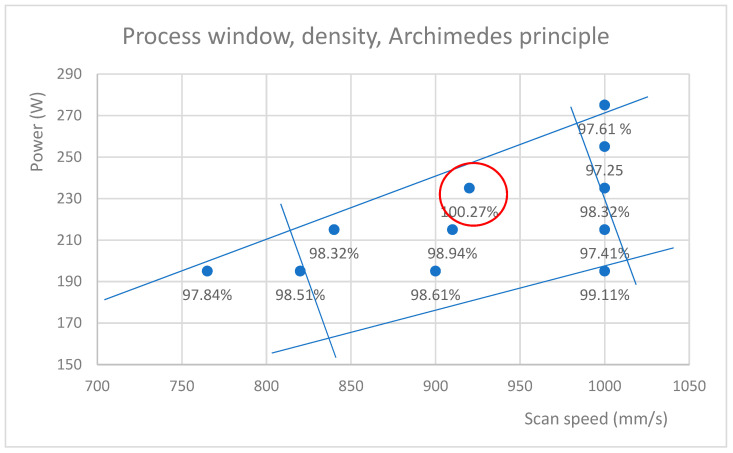
The highest densities, as determined by the Archimedes principle, were found at a VED level of 71 J/mm^3^ in process parameter C2 (235 W/920 mm/s). The red circle shows optimal density reached and blue lines are approximate process window borders.

The densities for each parameter set are plotted on the process window (Figure 12). Note that in the Archimedes principle, the densities are compared to a reference density—here, >7.97 g/mm^3^, as reported by the OEM—and, therefore, it is a relative density (percentage of the reference density) and can be higher than the reference. The highest density was found to be achieved with the parameter set C2 (VED of 71 J/mm^3^), which was also best for the visual inspection of the microscope images. In addition, C2’s “defect coefficient”—the number of defects multiplied by the average size—was the smallest of all the samples, alongside B3.

**Figure 13:**
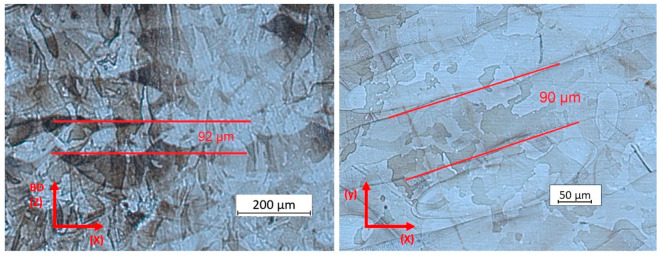
Parameter set C2′s micrograph side view (**left**) shows relatively deep melt pools. The bottom view (**right**) shows nicely organized melt pool tracks (etchant Beraha II).

The etched micrograph of C2, the highest density specimen, shows melt pool penetration (Figure 13) without severe keyhole formation and a melt pool depth of approximately 90 µm. The depth of the best-performing specimen C2 was deeper than the optimum penetration (~55 µm) suggested in the analysis of Gaikwad [38]. The melt pool width, mostly defined by the laser spot diameter, was measured to be 90 µm. The melt pool width/depth ratio was, therefore, 1.0. Referring to experiments performed on the Inconel 718 ratio, this low value indicates that the process is on the edge of transitioning from conductive mode to keyhole [39,40]. For 316 L, Zhang et al. measured a similar ratio when the scan speed was 870 mm/s but with slightly higher laser power, i.e., 260 W, and a 50 µm layer thickness [41]. The conclusion is that the specimen C2 parameters—235 W and 920 mm/s—are very close to the process window’s upper edge. 

**Figure 14:**
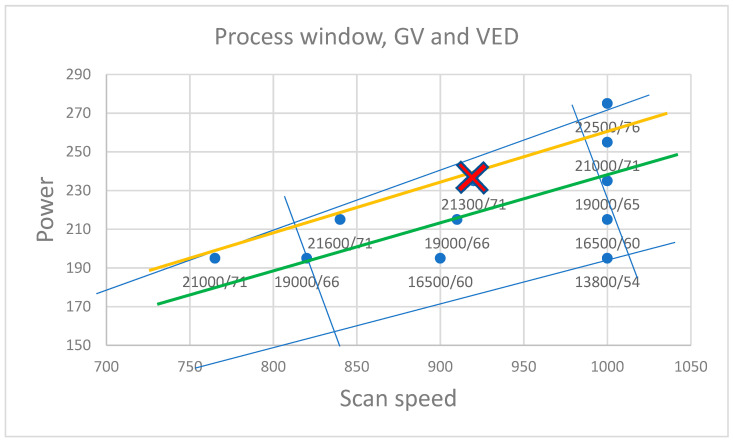
The optimal parameter set is close to the upper limit of the conducting melt area. The yellow line represents the upper threshold value, and the green line represents the lower. Blue lines approximate the process window.

C2’s VED was 71 J/mm^3^, and the respective GV emission was 21,268 GV, which is close to the upper threshold discovered in this study. The largest defect in set B samples for C2 was 709 px, which is comparable to a spherical defect diameter of 30 µm. While this could be classified as a critical defect, it is the only one found in the three images taken from the specimen. In Figure 14, the optimal density parameter is marked with a red cross, including the corresponding grey GV and VED. As can be seen, the optimum result is achieved at the upper limit of the conductive melting area, near the threshold found for this material. The yellow line represents the threshold for set A, and the green line represents set B.

## 4. Discussion

This study attempted to discover ways in which process monitoring—Optical Tomography—can be used to help engineer parameters to solve quality or printability issues that arise in some features of an arbitrary component. These features can include a rapidly changing layer area, sensitive features, hourglass shapes, and low-angle downskins that react differently to the exposure values. Moreover, features prone to large deformations during the process or critical features regarding fatigue strength that require defect-free structures might need specially engineered parameters. From the literature [12,22], it is known that defects link directly to lower mechanical properties, including fatigue or tensile properties. Therefore, in this study, the focus was on finding a link between defects and OT data rather than evaluating mechanical properties.

Laser exposure, which OT monitors, is a combination of laser power, scan speed, hatch distance, and layer thickness, and varying these parameters has multiple—even conflicting—influences on the print result. For example, a high scan speed results in fast cooling, which affects the evolution of the microstructure and also reduces the heat input unless it is compensated for with higher laser power, which can lead to keyhole porosity. In addition, hatch distance should be in synchronization with melt pool width and depth, defined by layer thickness, scan speed, and laser power, to enable full solidification. Thus, each parameter set is a multivariable compromise between productivity and material properties [24].

Evidently, there is a connection between the laser exposure energy used and the emission energy detected by OT. Moreover, based on the literature [12], process values can be linked to certain defects. A high laser exposure deepens the melt pool and increases the trapped gas pores, a low laser exposure causes inadequate melting and, thus, a lack of fusion, and a high scanning speed causes balling. However, for parameters close to optimal and inside the process window, the case is different. The differences in density and types of defects are so small that no bulletproof link between GVs and defects can be expressed, at least within this study.

In this study, the design of the experiment included a few parameters that resulted in identical VEDs but were composed of different laser power and scan speed combinations (Table 1). The data show that even if the VEDs are identical, the material quality is different depending on the parameter combination. For example, the parameters A3, B2, and C1 have the same VED, but sample C1 has many more defects. Based on the literature, the same effect can be assumed to exist when varying other VED parameters—that is, hatch distance and layer thickness. An important note is that materials have different absorption and reflection rates and emit energy in different amounts. Therefore, all parameter engineering must be performed based on material-specific data.

The data used for the parameter engineering were numerical GV data, not the image data usually considered to be the output of the OT system. The use of the image data can take advantage of machine interpretation, possibly with the aid of artificial intelligence, as reported by Akmal [42]. This can reveal “defect-based spots” for detailed analysis of local pixel GVs that can even allow for real-time process tuning.

The main finding of this study is the localized process window threshold, after which the defect size tends to increase from minor to critical. This threshold was found to be 19,000–21,000 GV for 316 L. For reference, similar samples printed with a process developed by OEM and provided as default parameters for 20 µm layer thickness typically show average GVs of 13,000 GV. Emulating normal conditions, the GVs stay below 15–16,000 GV. In engineering, the avoidance of critical defects is of the utmost importance, especially for components under fatigue stress. Therefore, scanning the numerical GVs for values exceeding the threshold can help manufacturers avoid component failure and choose the correct values for laser exposure.

Here, the defects were evaluated from the micrographs using optical microscopy. However, it may be more effective to analyze defects using X-ray computed tomography similar to Nafar [22], which can reveal the internal defects in the specimen in its entirety, considering the resolution limitations placed on the size of the specimen. This could possibly give a more accurate threshold limit. It must also be noted that the threshold limit discovered in this study is likely to be material-specific as well as dependent on layer height due to differences in laser energy absorption and reflectivity.

The critical size was considered to be 20 µm based on the literature. Therefore, in set B, all defect areas under 10 px in size were excluded. The number of defects was inversely proportional to the size of defects for those samples that exceeded the threshold limit—if the number of defects was high, the size was low, and vice versa. The reason for this was not clear. Possibly, a high-energy melt pool with a stable keyhole penetration produces, in general, better material quality by repairing possible defects through re-melting and deeper penetration. However, it is prone to random collapses that have more severe consequences—for example, larger defects. This should be studied further, for example, by using a high-speed camera or simulations.

While OT statistical data cannot fully solve parameter engineering problems when detailing the process inside the process window, analyzing it can help manufacturers avoid the most critical structural defects. The averaged layer data, which the OT statistic represents, do not consider events within a single layer; therefore, a more accurate OT analysis using a threshold limit should focus on sections of the build and on problematic feature-specific layers. This situation can emerge, for example, in hourglass geometries, where local heat accumulation and low heat conductivity affect thermal conditions and, thus, defect formation. Image processing and artificial intelligence can be of assistance in evaluating the OT raw data, which also considers local defect-prone areas of a layer. As a whole, the subject—component-specific process engineering through OT process monitoring—needs more research focusing on the behavior of different features, for example, downskin topography, the rough quality on down-facing surfaces, or narrow beams and thin walls.

## 5. Conclusions

Broadly speaking, digital manufacturing, which includes AM, requires new tools and workflows to operate most efficiently. These tools include both software and hardware equipment that monitors new processes and provides data and information about them. Future efforts in AM development should focus on controlling and adjusting the process. In conclusion, the following points can be made:

- Process parameters, including laser power and scan speed, define material properties and can be monitored via OT. The correlation between VEDs and GVs was complete correlation with a value of 0.99.
- High exposure parameters—here, D1 and E1 (255 W, 1000 mm/s and 275 W, 1000 mm/s)—define a boundary where material turns from dense to include defects typical for keyhole and balling regimes. Similarly, a low density achieved with lower WEDs assigns a lower bound.
- There is a threshold for GVs, after which the defect size increases. Defect size was found to increase significantly at 19,000 GV, but it increased more slowly below this threshold.
- The number of defects decreased and the defect size increased when VED was adjusted to be higher by slowing the scan speed, as shown in Table 2.
- The importance of parameter engineering is vital for novel applications. Therefore, more work regarding the use of process monitoring in the development of process conditions is needed to enable component-specific processes.

Optical tomography is one of several ways to monitor the AM process. Other technologies include melt pool monitoring, powder bed imaging, and ultrasound monitoring. These systems produce high amounts of data from process events. Future work should focus on merging these data sources. Artificial intelligence can potentially enable the automation of real-time defect correction if process anomalies are detected since a defect can be healed during the following two to three layers. Such work is already in progress [42,43,44]. Printing three layers takes, for example, five minutes, leaving time for the direct adjustment of parameters like laser power, but the time is hardly enough to re-calculate and re-upload the whole build.

In addition, more information about the impact of the exposure value on component-specific, support-free builds and problematic features is needed to enable agile parameter engineering. For example, current research mainly covers varying only the infill parameters, while research about contour parameters affecting surface quality is inadequate. This would be of great importance for developing parameters for fatigue-critical structures. Optical Tomography has the advantage of monitoring the process’s thermal behavior even during the print. Given the possibility of adjusting equipment during a build, OT has the potential to solve printability issues.

## Figures and Tables

**Table 1:** Process parameters were chosen based on different compositions of VED. Laser power and scan speed were modified, and hatch distance and layer thickness were constant. VED varied between 54.2 J/mm^2^ (A1) and 76.4 J/mm^2^ (E1). A–E represent laser power and 1–4 represent scan speeds.

	A	B	C	D	E
1	195 W/1000 mm/s	215 W/1000 mm/s	235 W/1000 mm/s	255 W/1000 mm/s	275 W/1000 mm/s
	VED 54.17 J/mm^3^	VED 59.72 J/mm^3^	VED 65.28 J/mm^3^	VED 70.83 J/mm^3^	VED 76.39 J/mm^3^
2	195 W/900 mm/s	215 W/910 mm/s	235 W/920 mm/s		
	VED 60.19 J/mm^3^	VED 65.63 J/mm^3^	VED 70.95 J/mm^3^		
3	195 W/820 mm/s	215 W/840 mm/s			
	VED 66.06 J/mm^3^	VED 71.10 J/mm^3^			
4	195 W/765 mm/s				
	VED 70.81 J/mm^3^				

**Table 2:** Sizes and numbers of defects larger than 10 px in the set B specimen.

	A1	A2	A3	A4	B1	B2	B3	C1	C2	D1	E1
Average size	101	138	276	269	109	447	214	90	39	113	167
Number of defects	149	109	62	50	213	54	31	178	140	228	271

## Data Availability

The raw data supporting the conclusions of this article will be made available by the authors upon request. Optical Tomography data are available in Björkstrand, Roy (2024), “Metal laser-based Powder Bed Fusion process development using Optical Tomography”, Mendeley Data, V1, https://doi.org/doi:10.17632/t2y7hv5rzt.1.

## Supplementary Materials

The following supporting information can be downloaded at: https://www.mdpi.com/article/10.3390/ma17071461/s1, Table S1: Set B micrographs.
