# Supplementary material for: Metal Laser-Based Powder Bed Fusion Process Development Using Optical Tomography
Source: Materials (Basel). 2024 Mar 22;17(7):1461. doi: 10.3390/ma17071461 (PMC11012340; doi:10.3390/ma17071461)
Supplement: Supplementary file 1 [file materials-17-01461-s001.zip › Table S1 - micrograph images.pdf]

Table S1. Micrographs of set B samples. The defects were analyzed from three images covering 10% of sample area. Each image below represents a 2.68 mm × 2.23 mm area.

	**1st**	**2nd**	**3rd**
A1	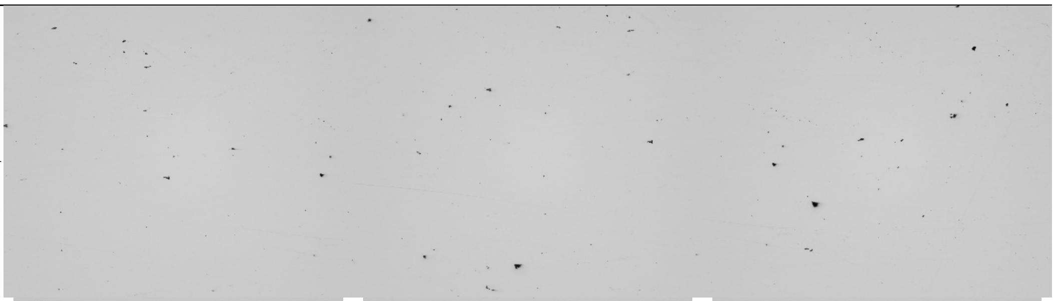		
A2	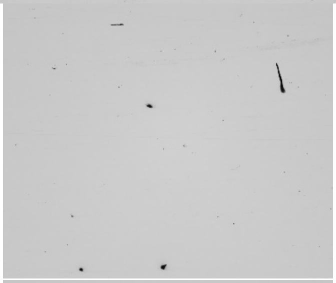	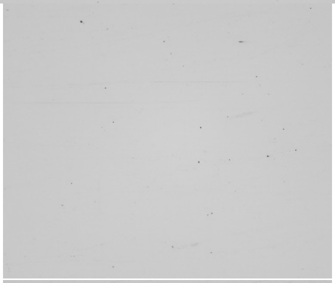	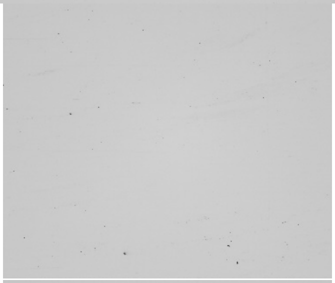
A3	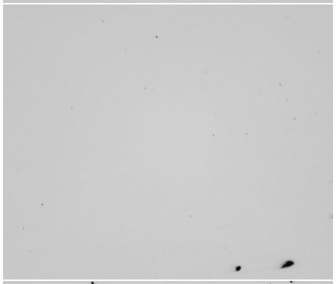	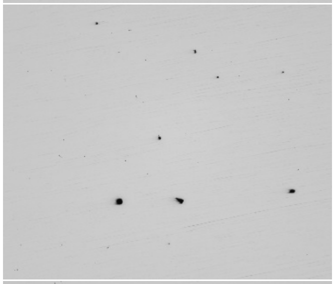	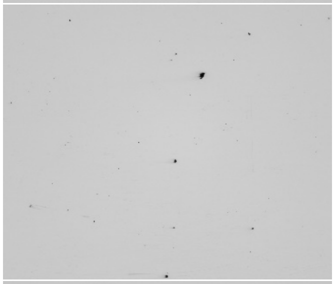
A4	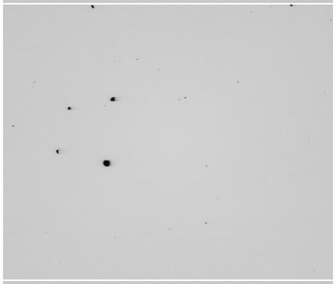	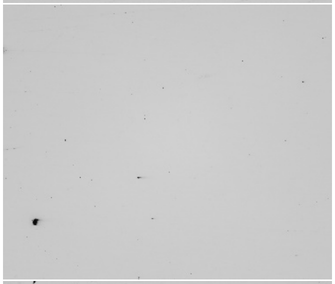	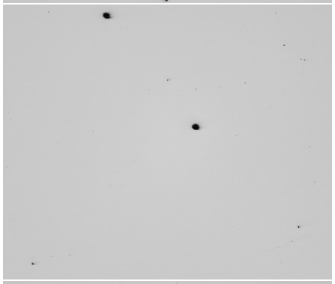
B1	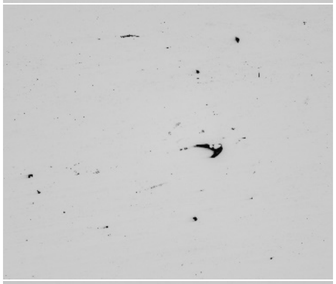	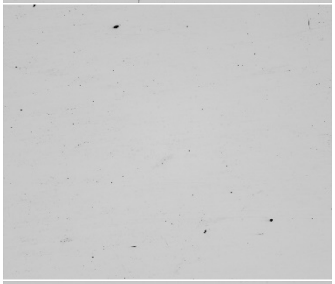	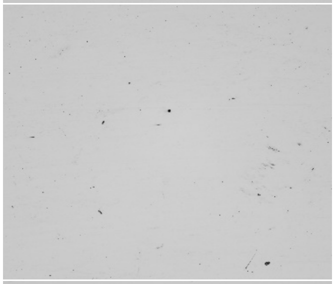
B2	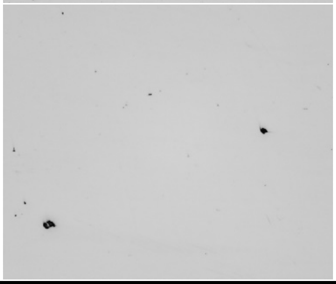	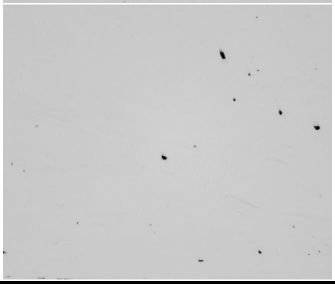	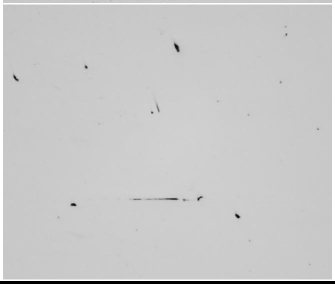

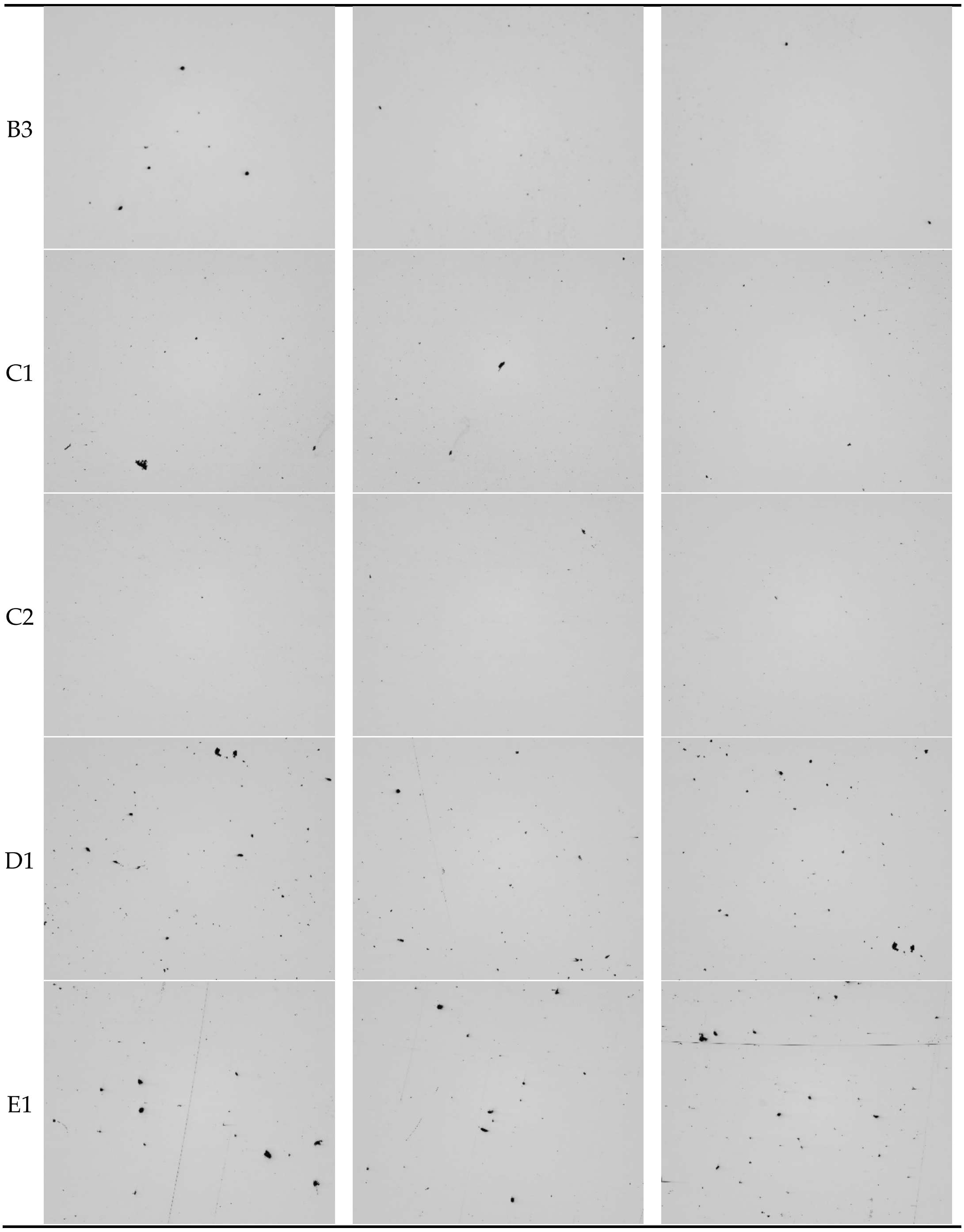

Set B micrographs, total magnification 3.15.
