# Supplementary figures and images for: Metal Laser-Based Powder Bed Fusion Process Development Using Optical Tomography
Source: Materials (Basel). 2024 Mar 22;17(7):1461. doi: 10.3390/ma17071461 (PMC11012340; doi:10.3390/ma17071461)

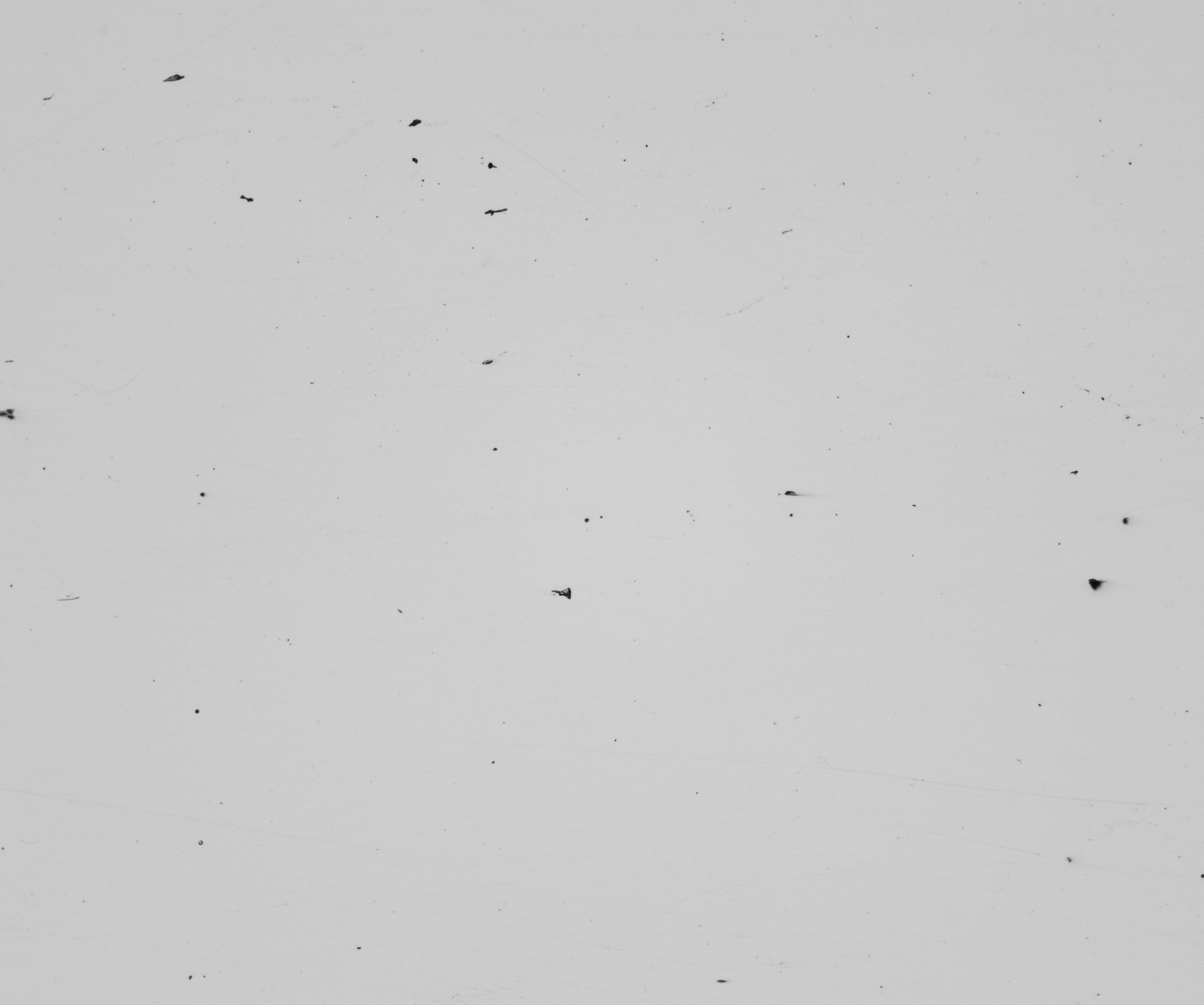

Supplement: Supplementary file 1 [file materials-17-01461-s001.zip › A1_2.jpg]

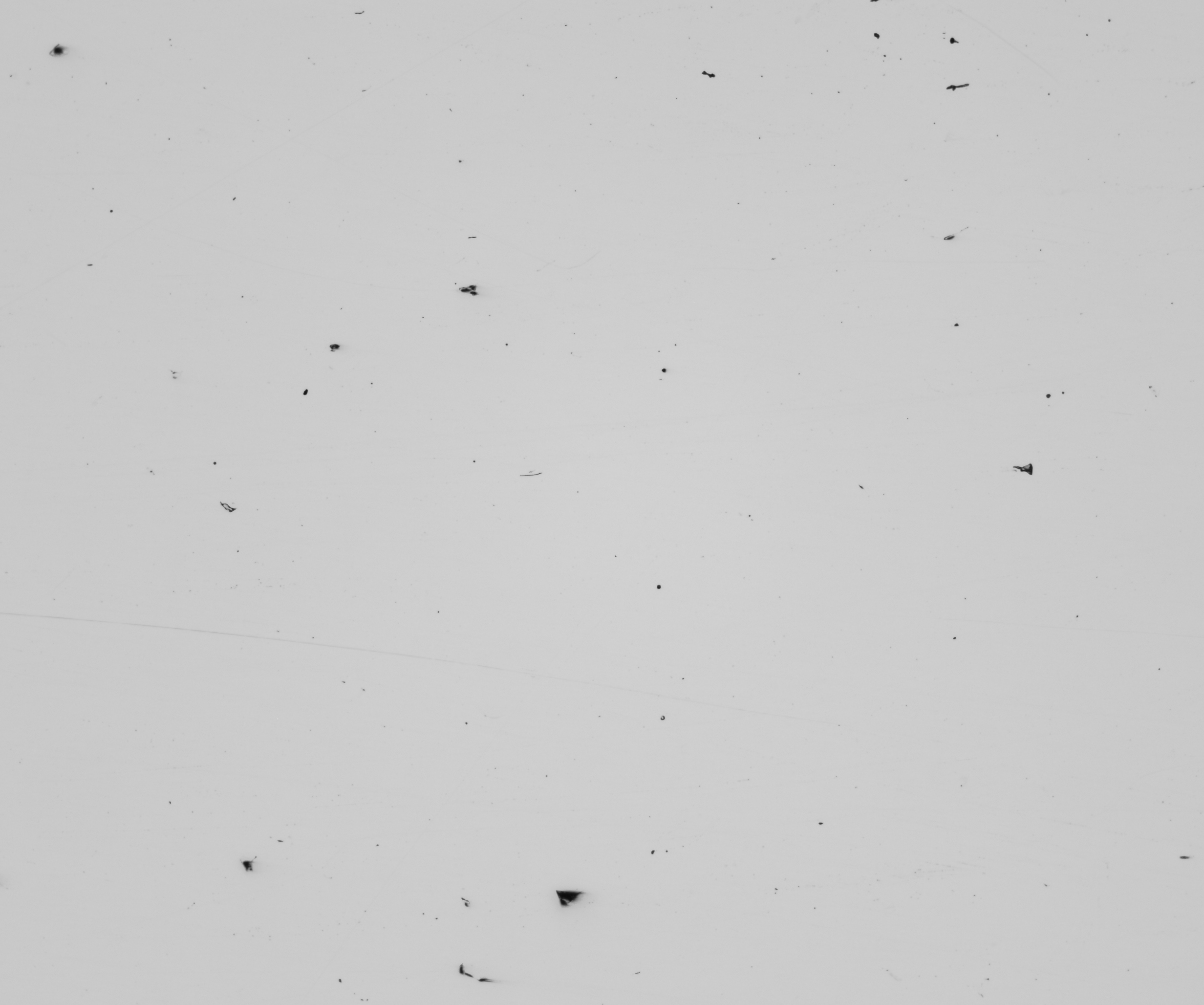

Supplement: Supplementary file 1 [file materials-17-01461-s001.zip › A1_3.jpg]

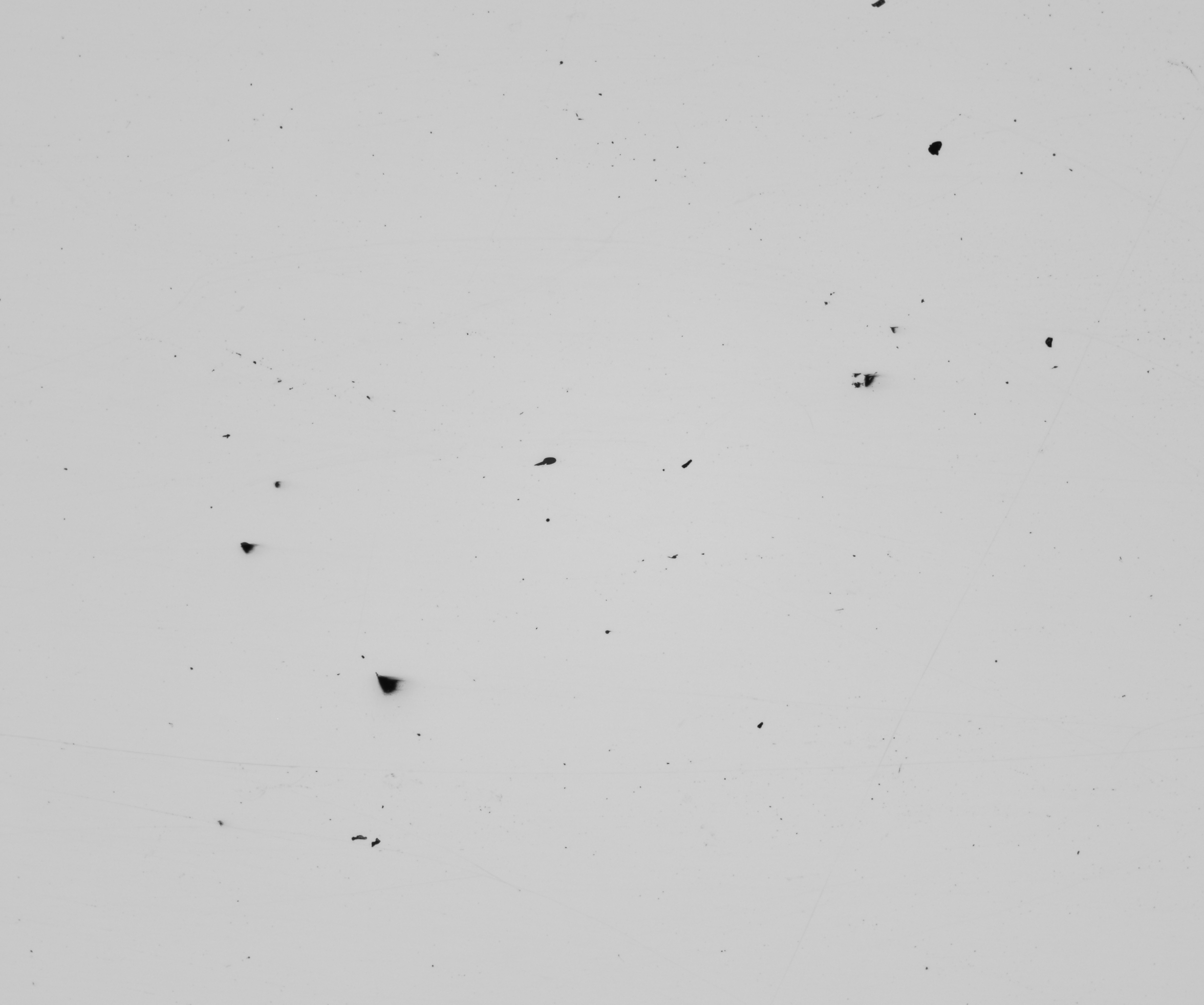

Supplement: Supplementary file 1 [file materials-17-01461-s001.zip › A1_4.jpg]

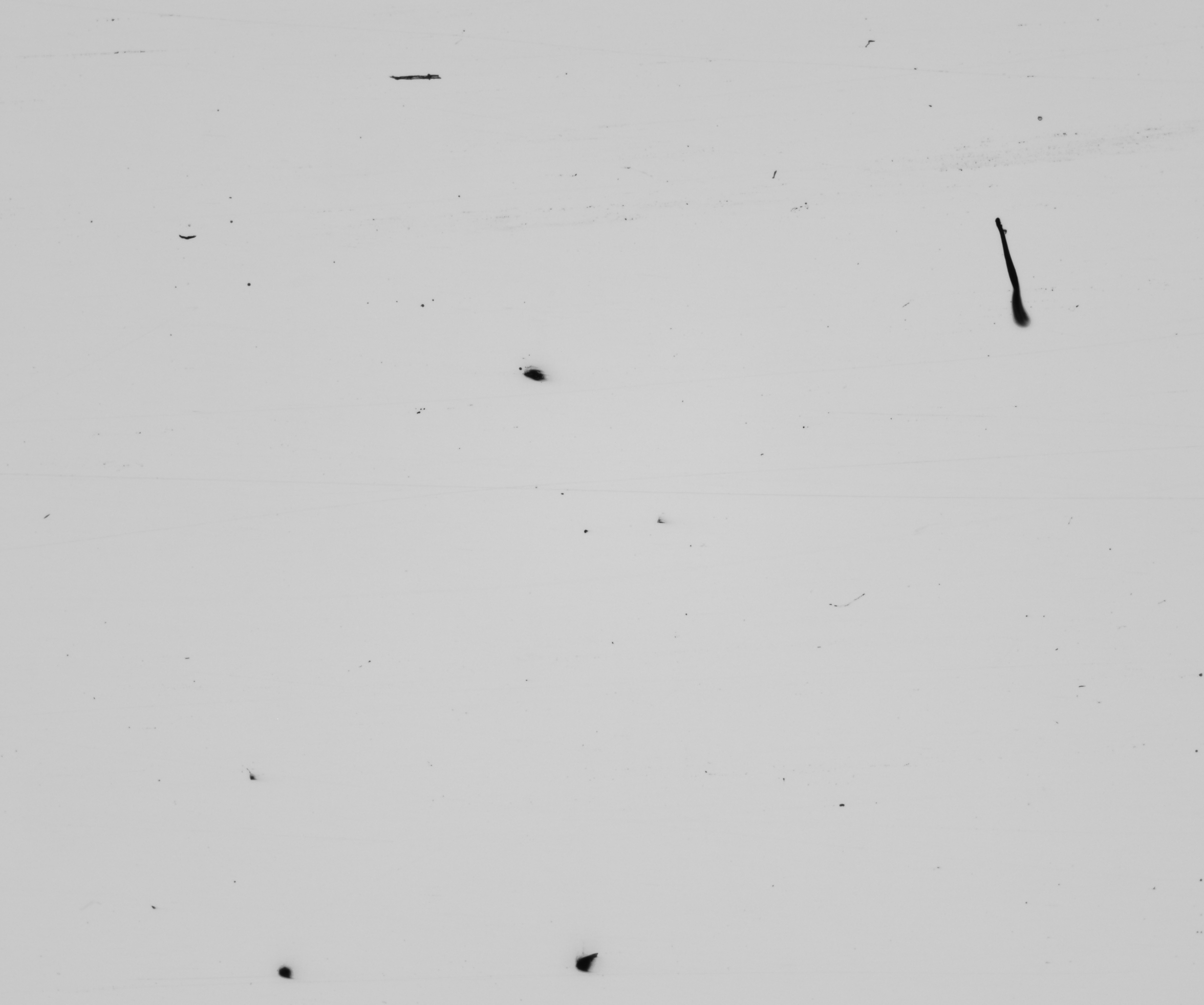

Supplement: Supplementary file 1 [file materials-17-01461-s001.zip › A2_2.jpg]

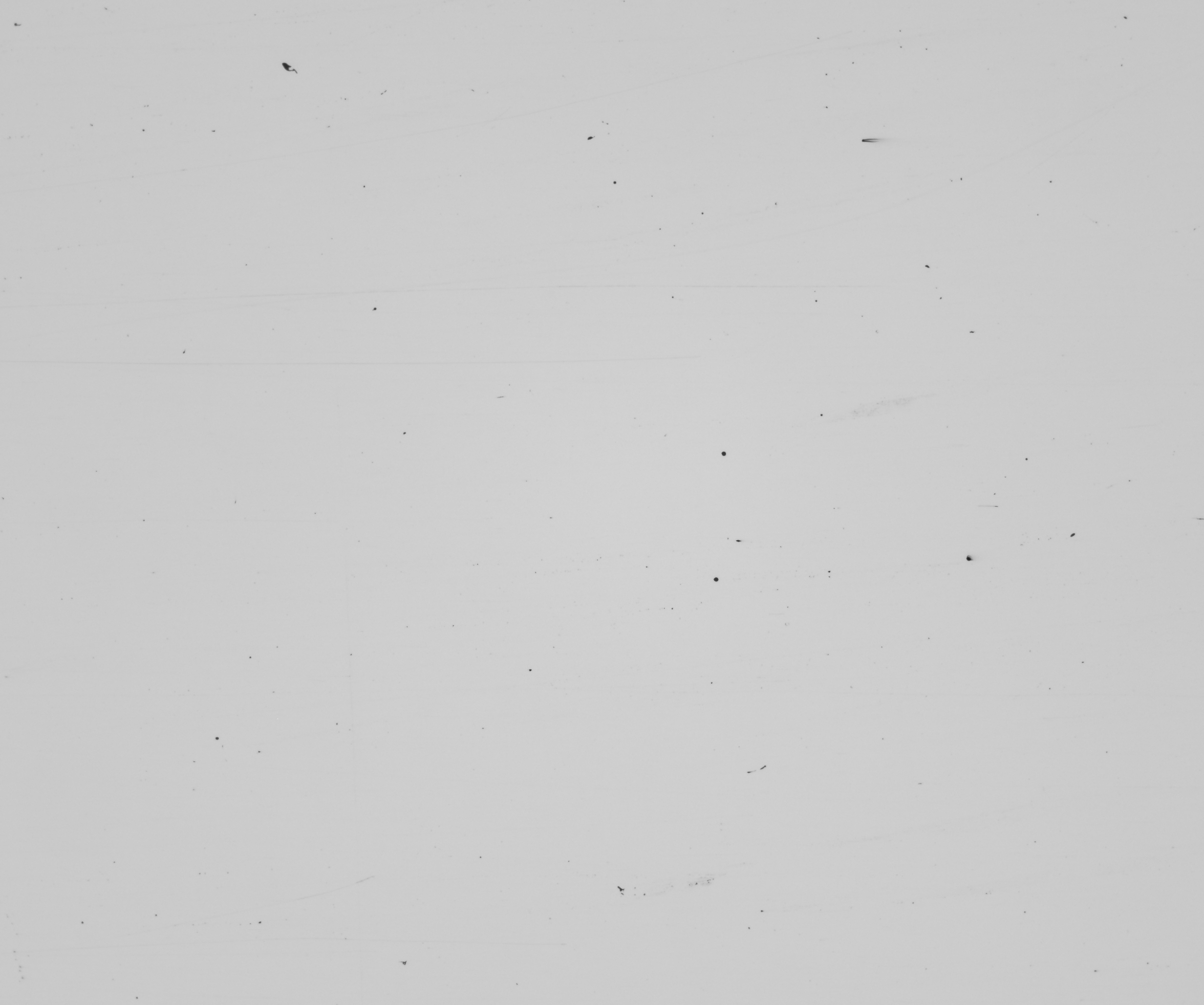

Supplement: Supplementary file 1 [file materials-17-01461-s001.zip › A2_3.jpg]

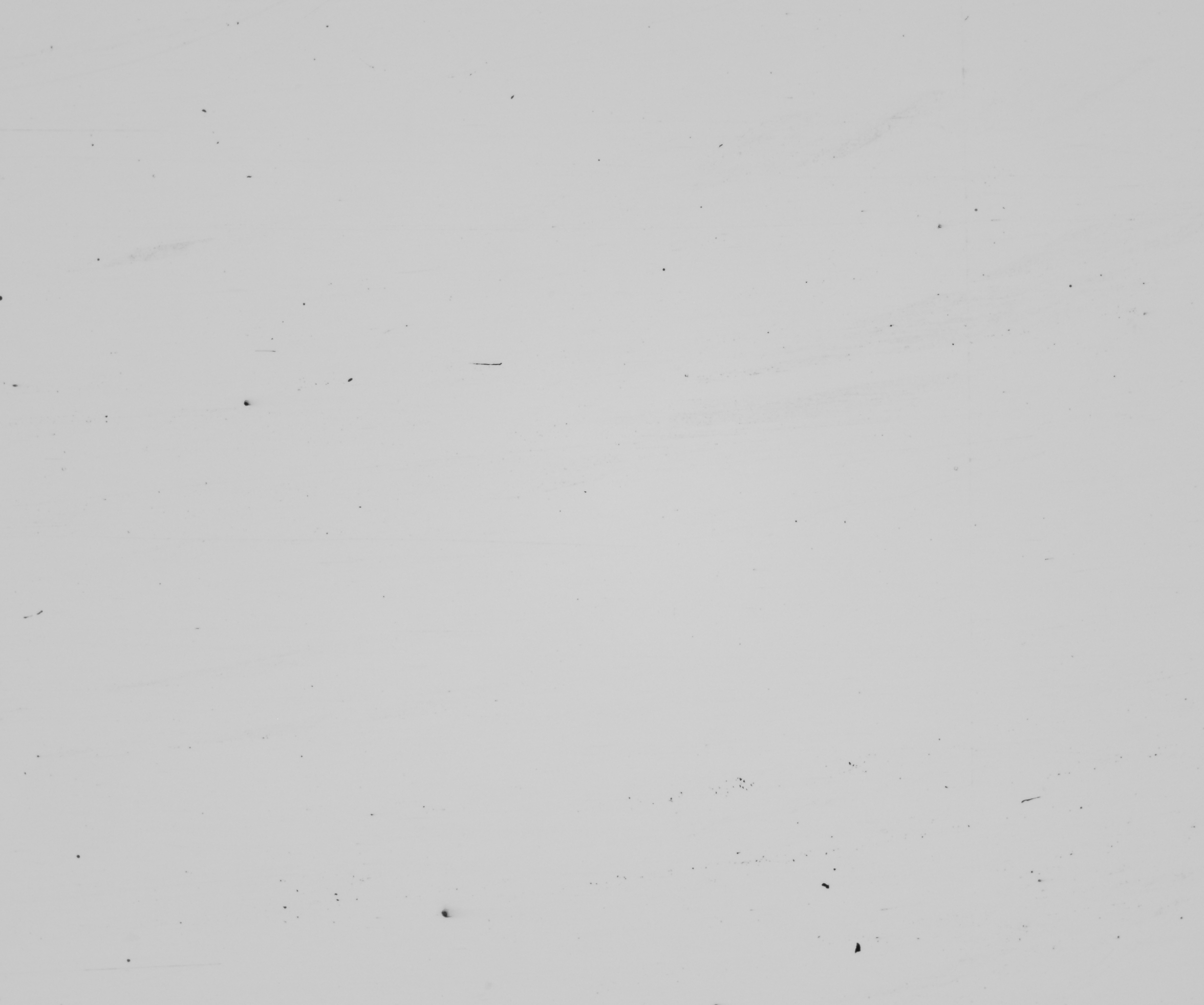

Supplement: Supplementary file 1 [file materials-17-01461-s001.zip › A2_4.jpg]

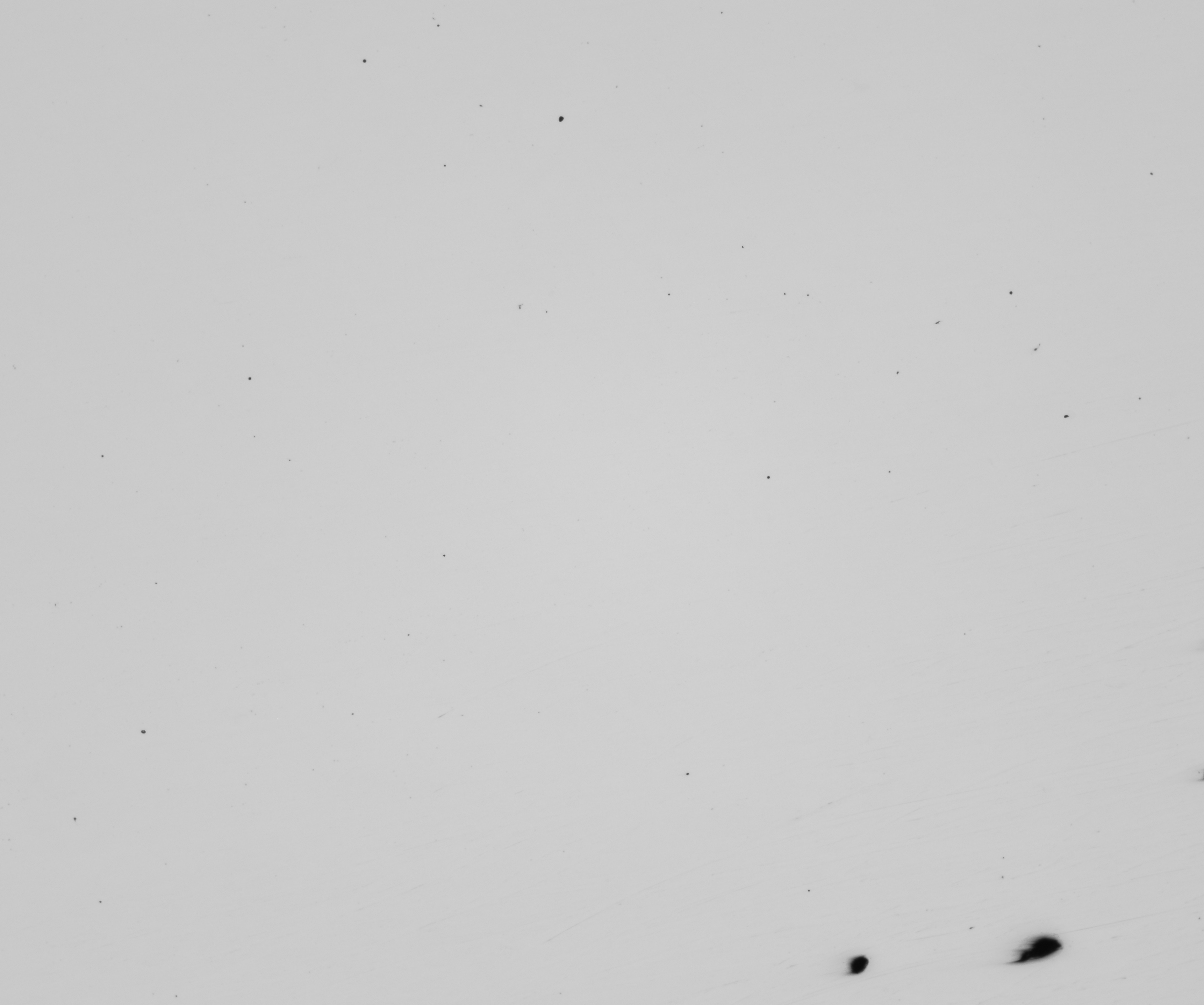

Supplement: Supplementary file 1 [file materials-17-01461-s001.zip › A3_2.jpg]

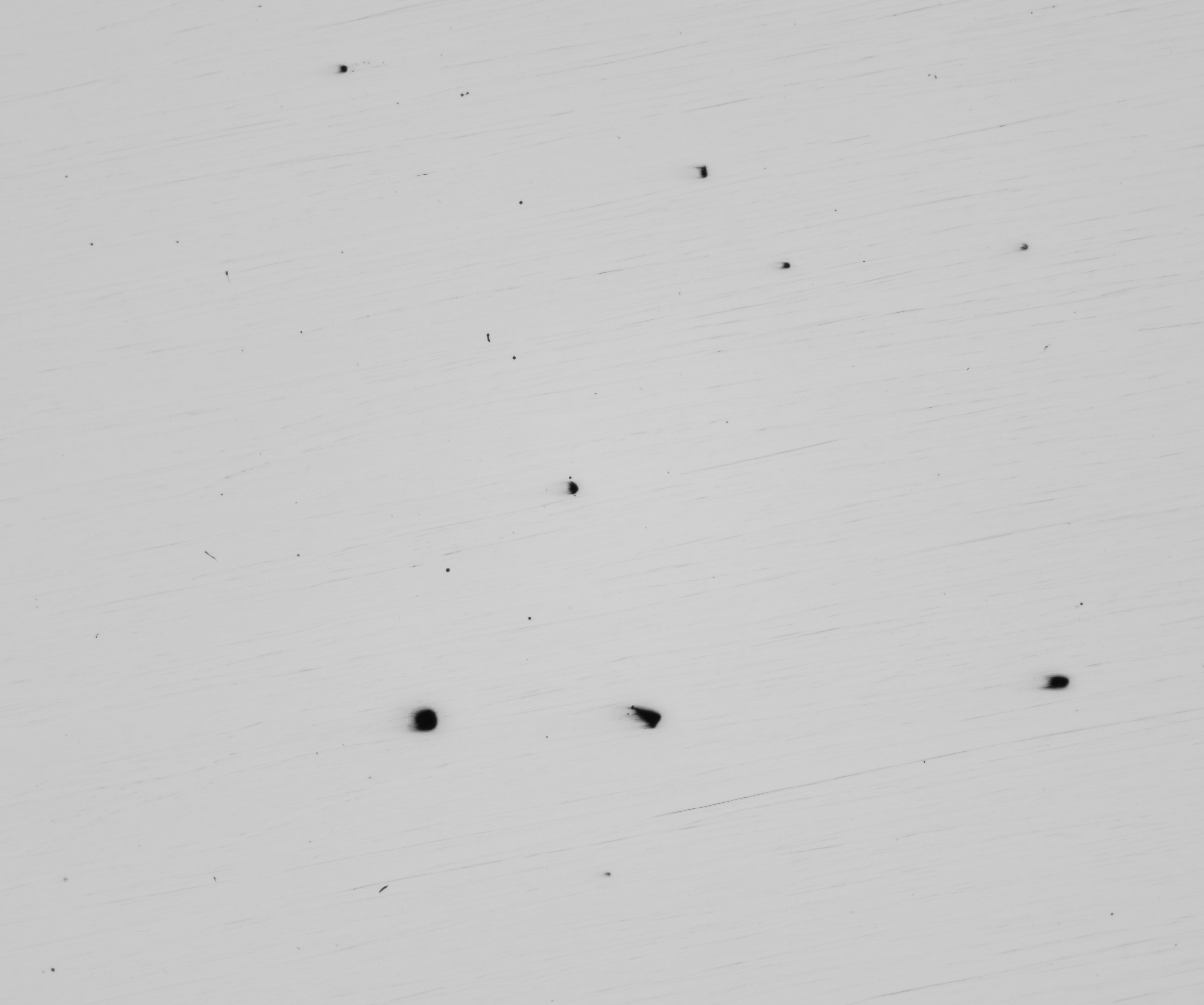

Supplement: Supplementary file 1 [file materials-17-01461-s001.zip › A3_3.jpg]

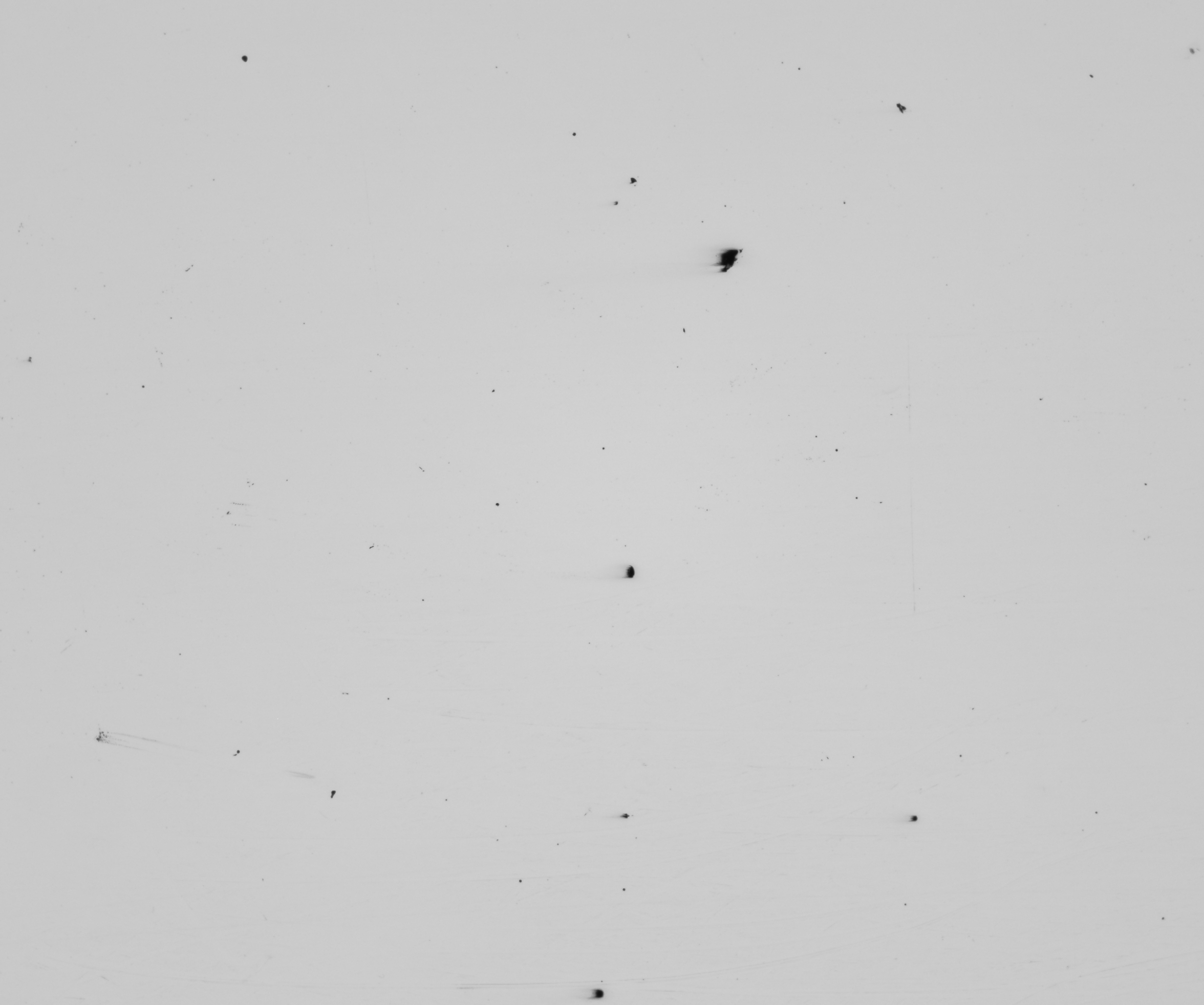

Supplement: Supplementary file 1 [file materials-17-01461-s001.zip › A3_4.jpg]

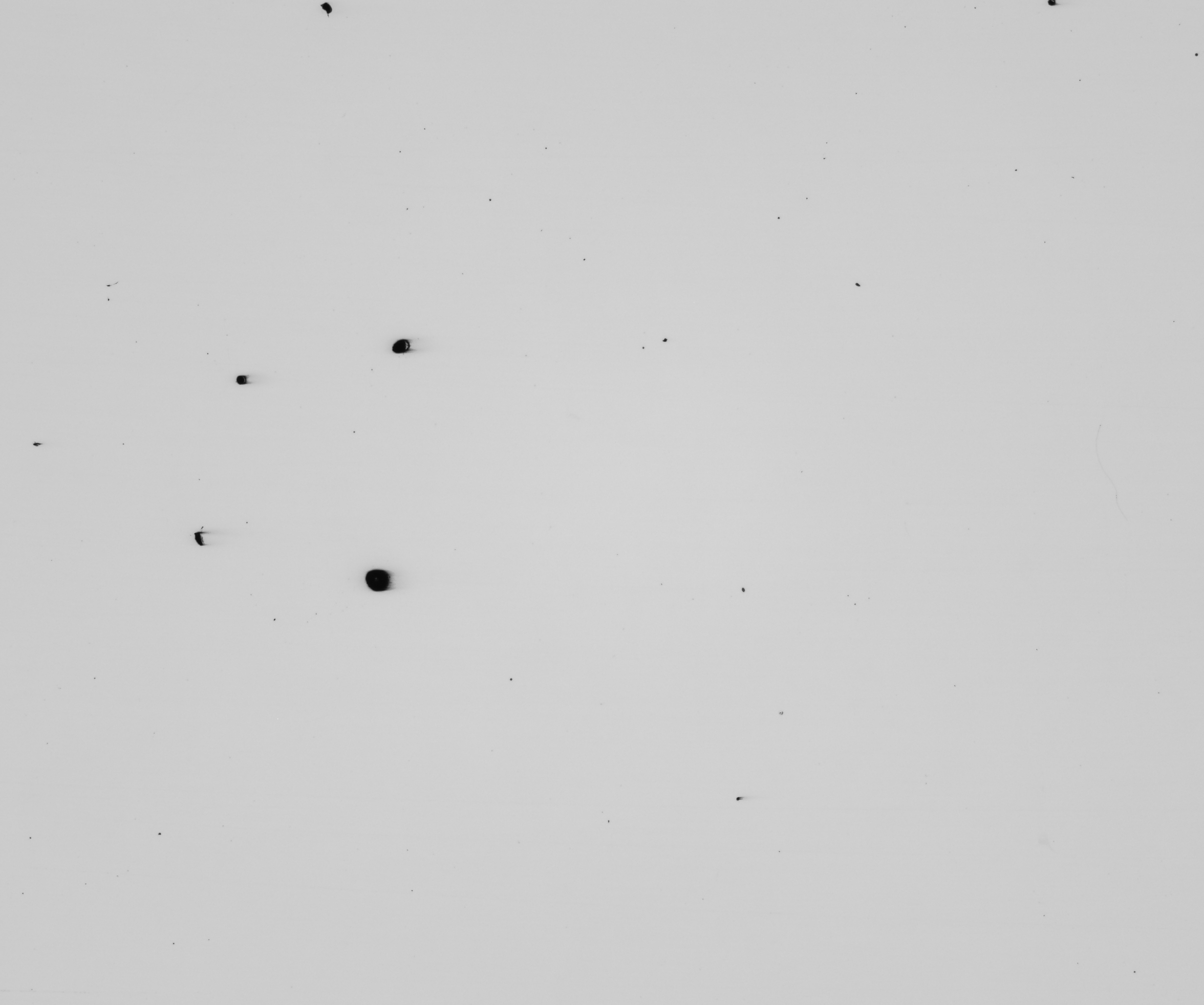

Supplement: Supplementary file 1 [file materials-17-01461-s001.zip › A4_2.jpg]

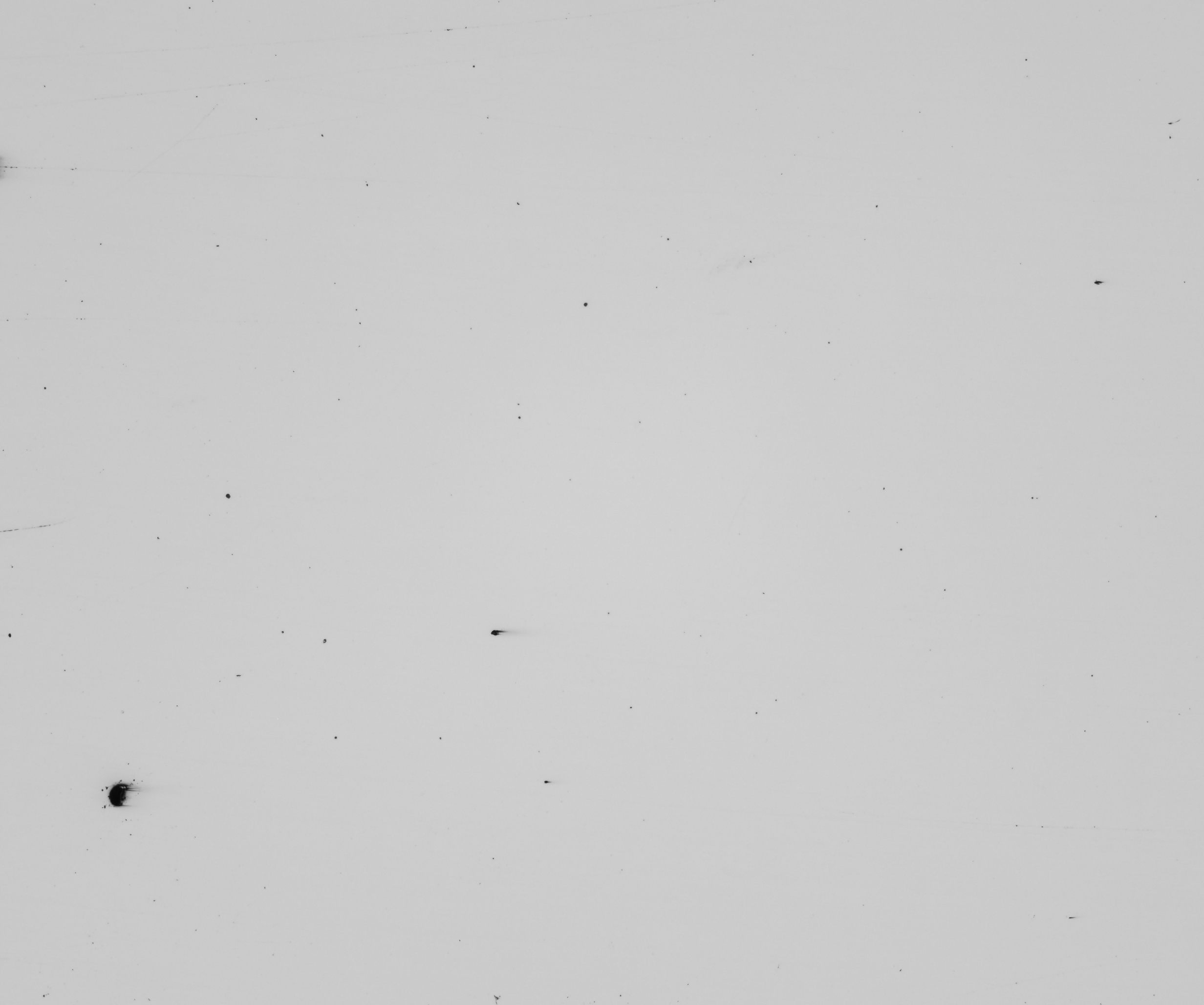

Supplement: Supplementary file 1 [file materials-17-01461-s001.zip › A4_3.jpg]

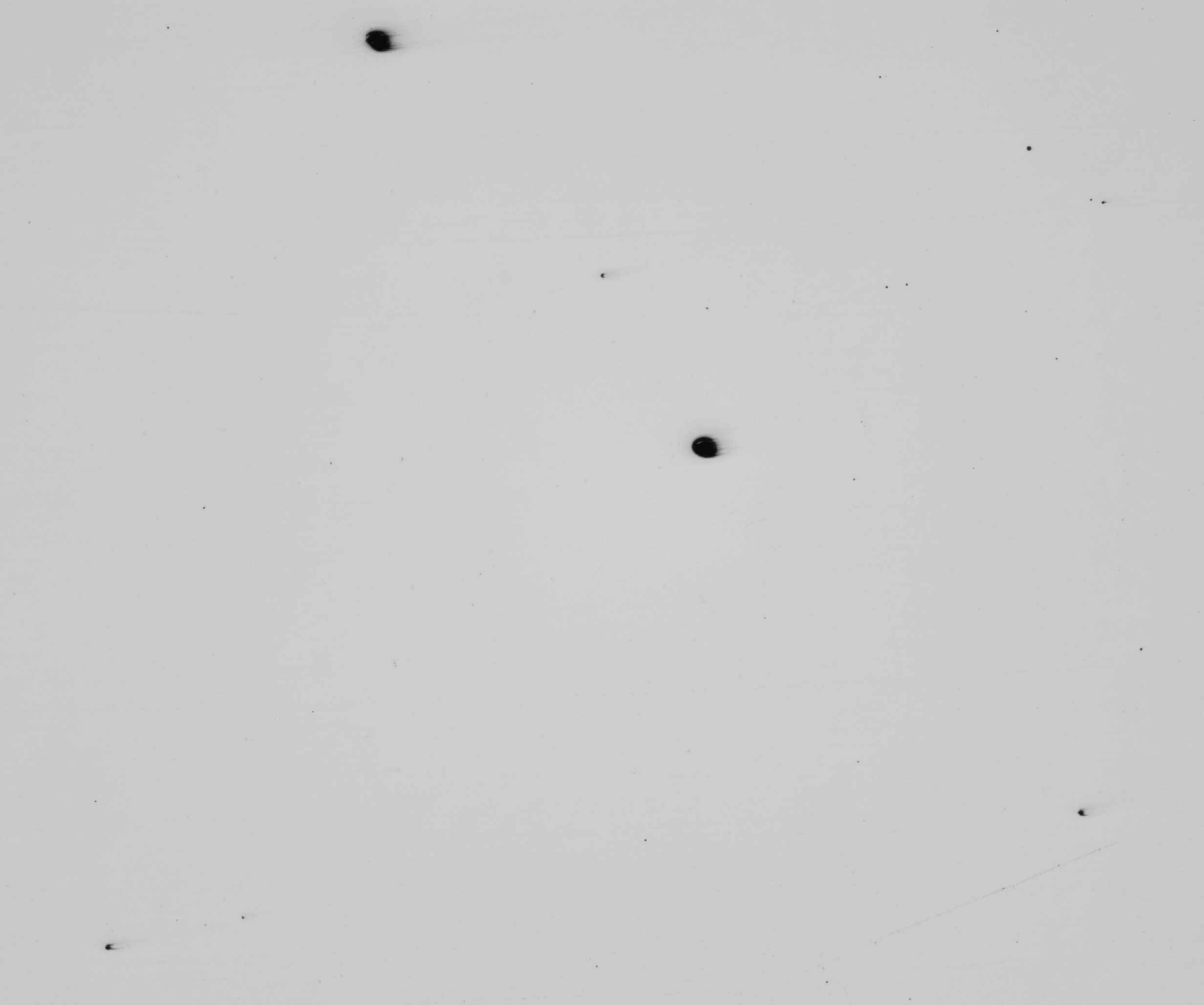

Supplement: Supplementary file 1 [file materials-17-01461-s001.zip › A4_4.jpg]

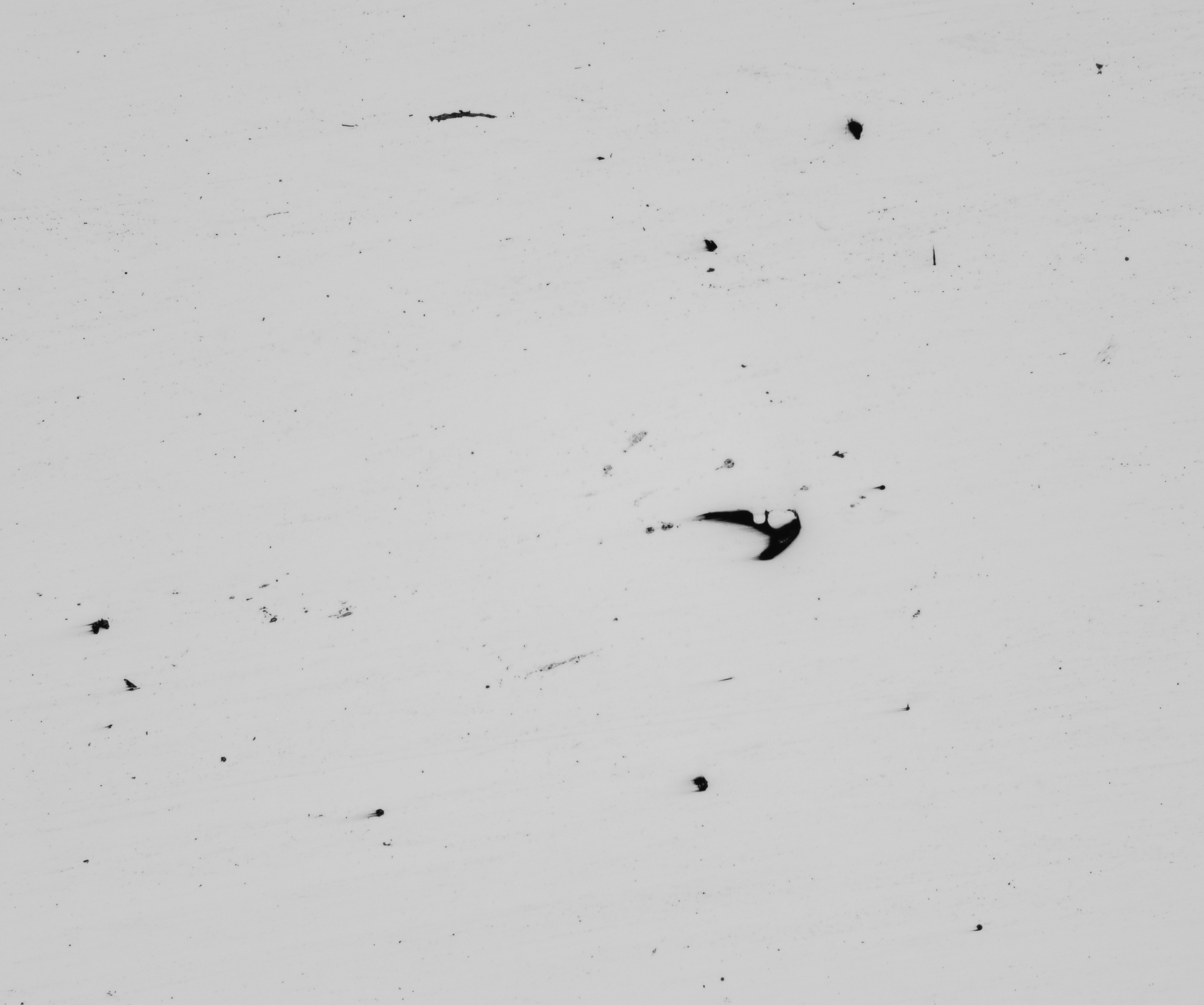

Supplement: Supplementary file 1 [file materials-17-01461-s001.zip › B1_2.jpg]

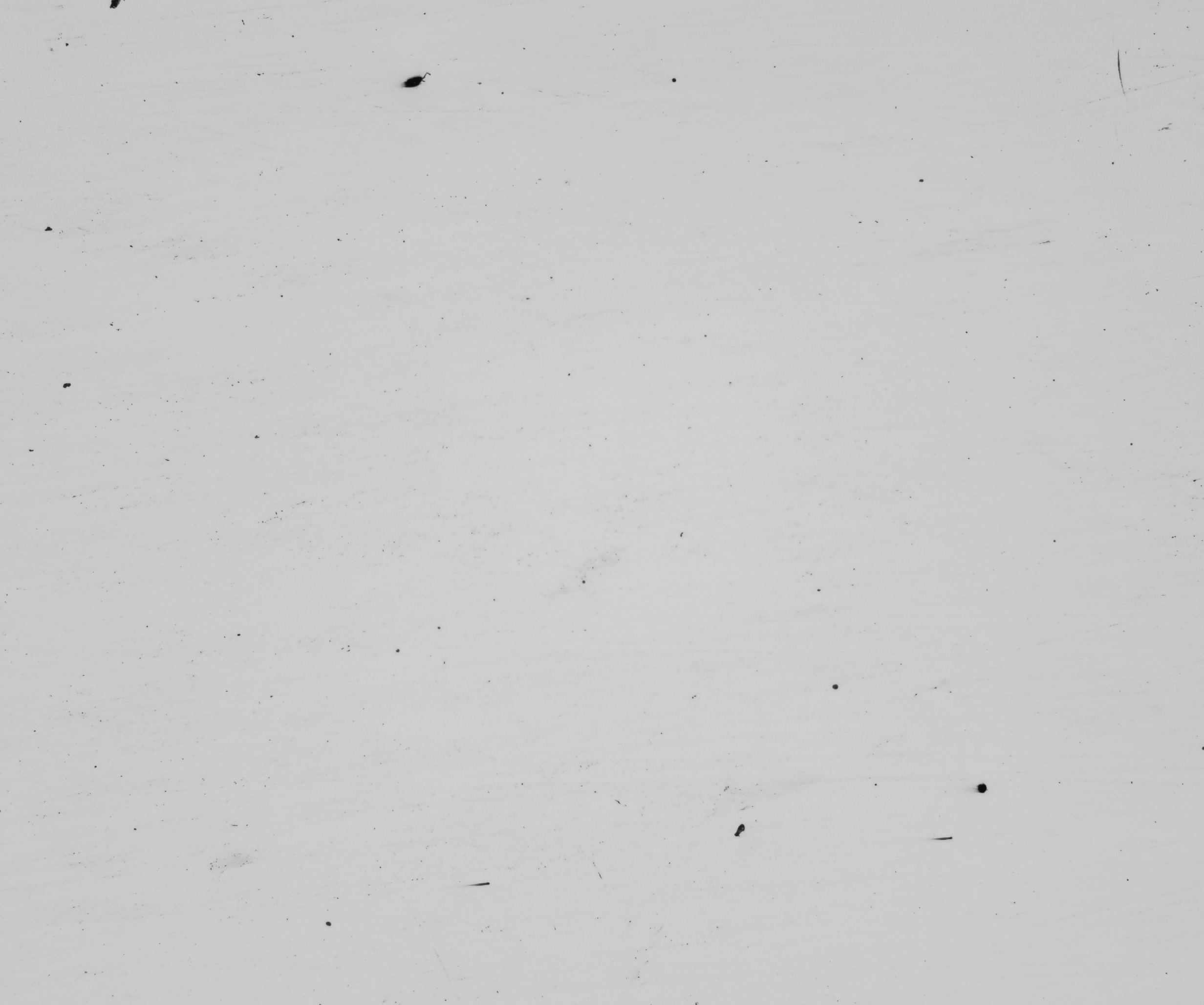

Supplement: Supplementary file 1 [file materials-17-01461-s001.zip › B1_3.jpg]

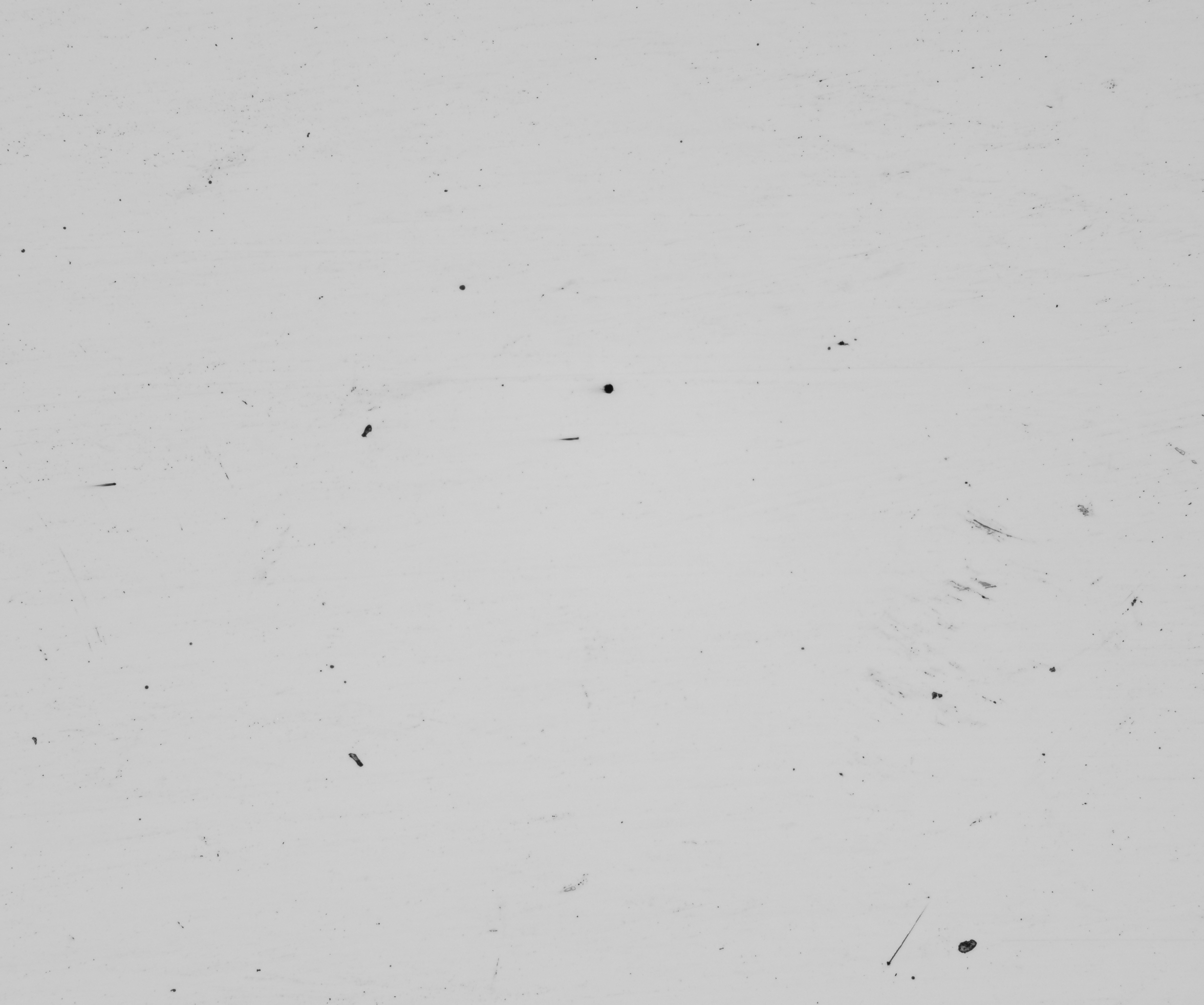

Supplement: Supplementary file 1 [file materials-17-01461-s001.zip › B1_4.jpg]

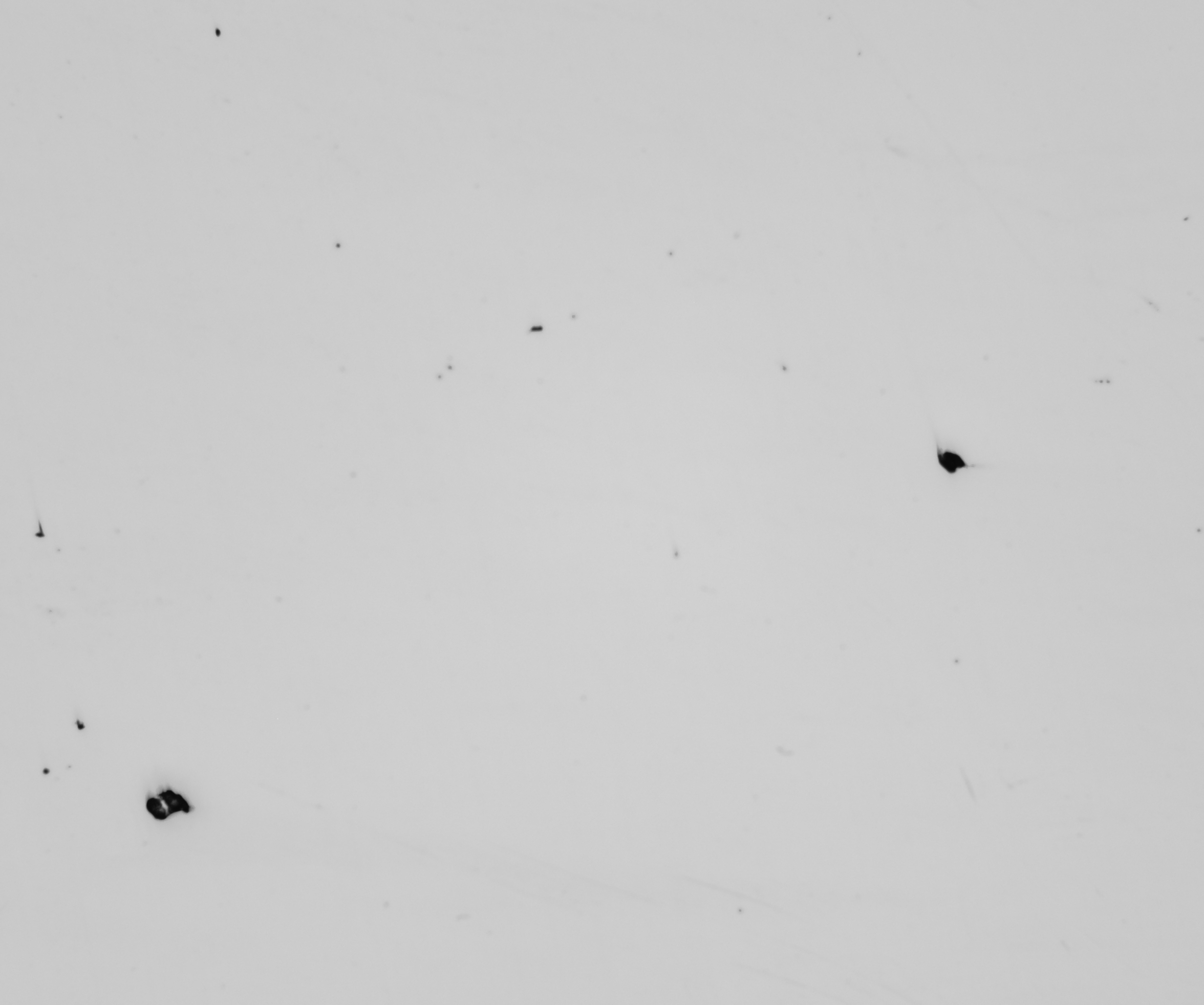

Supplement: Supplementary file 1 [file materials-17-01461-s001.zip › B2_2.jpg]

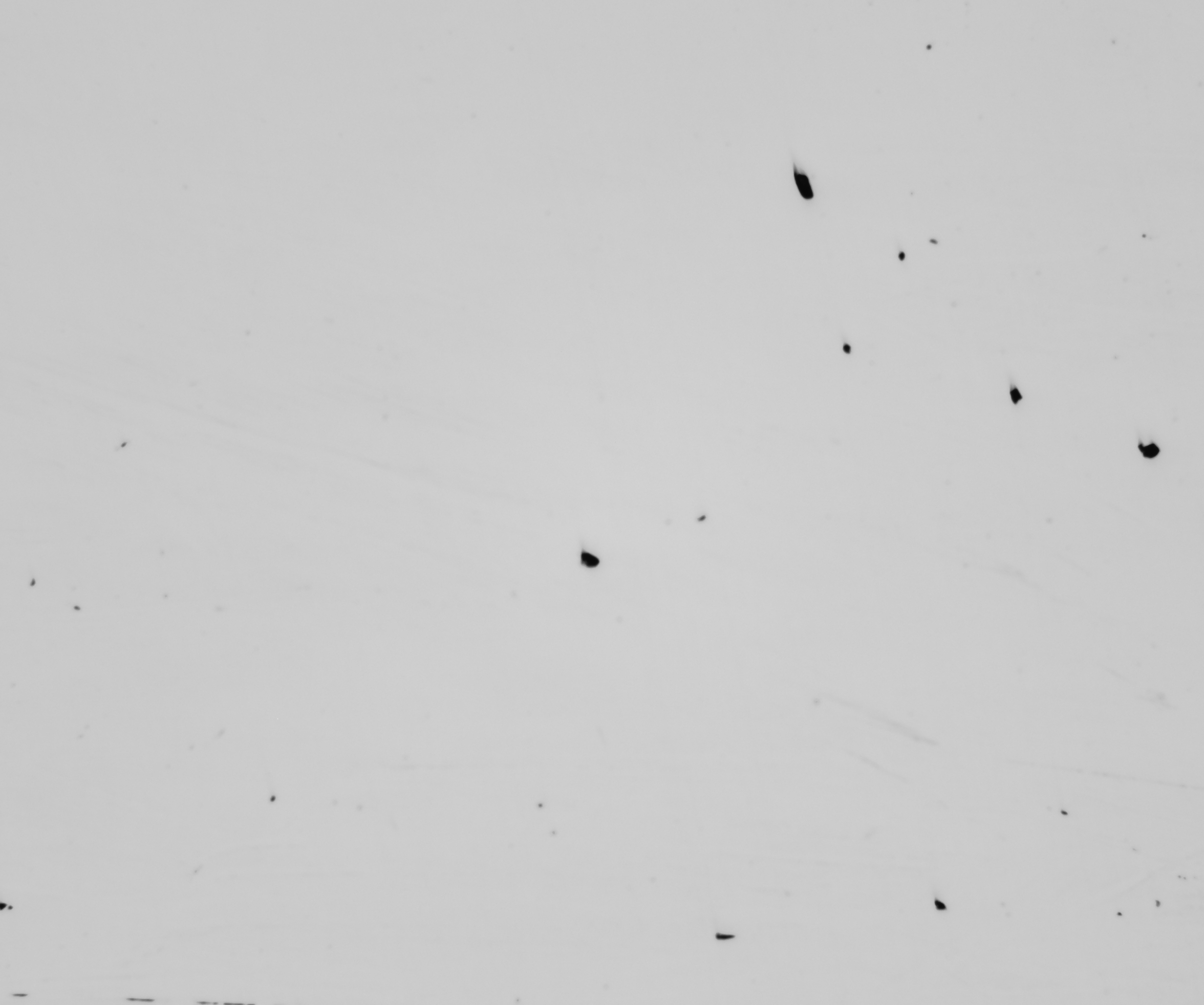

Supplement: Supplementary file 1 [file materials-17-01461-s001.zip › B2_3.jpg]

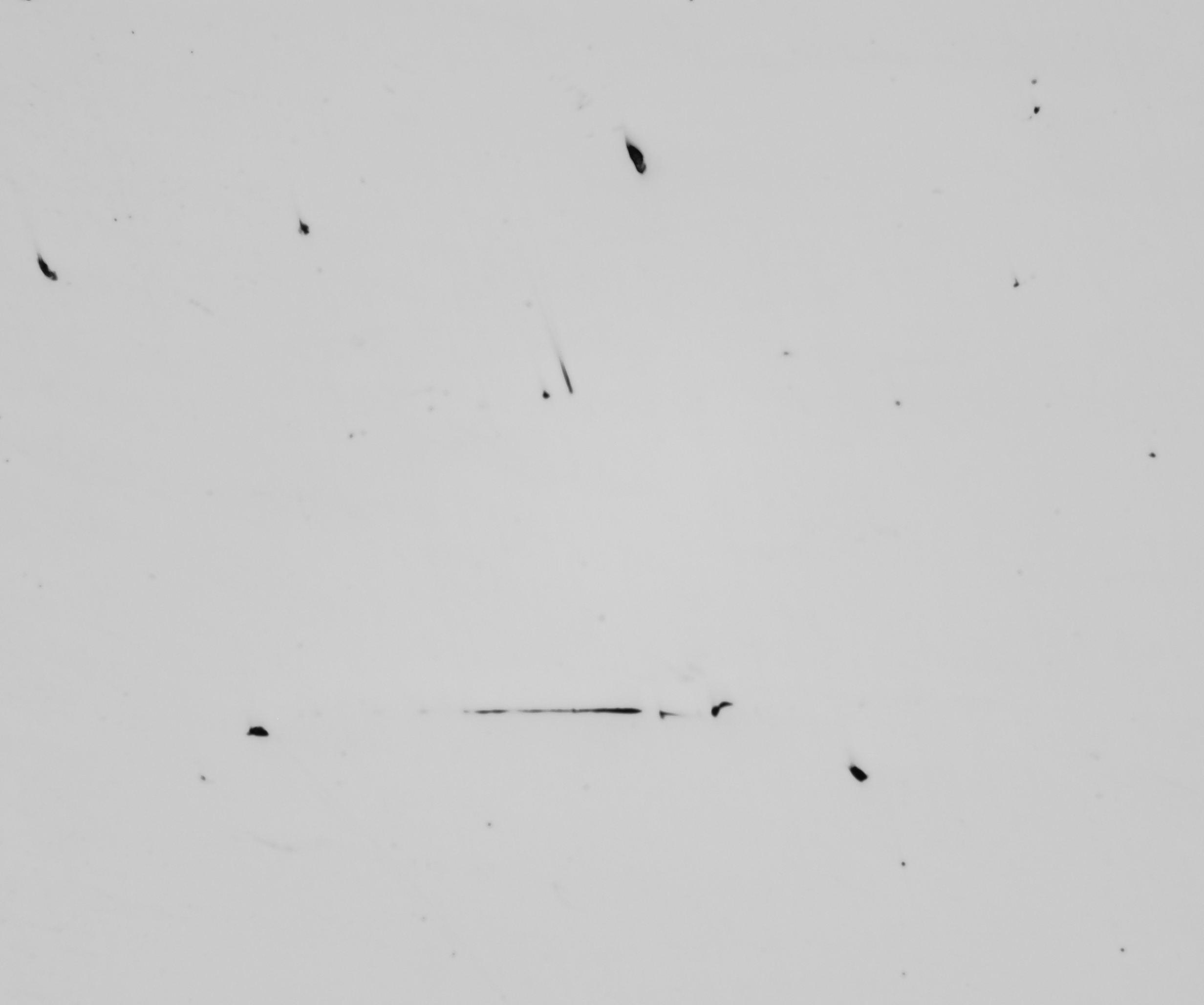

Supplement: Supplementary file 1 [file materials-17-01461-s001.zip › B2_4.jpg]

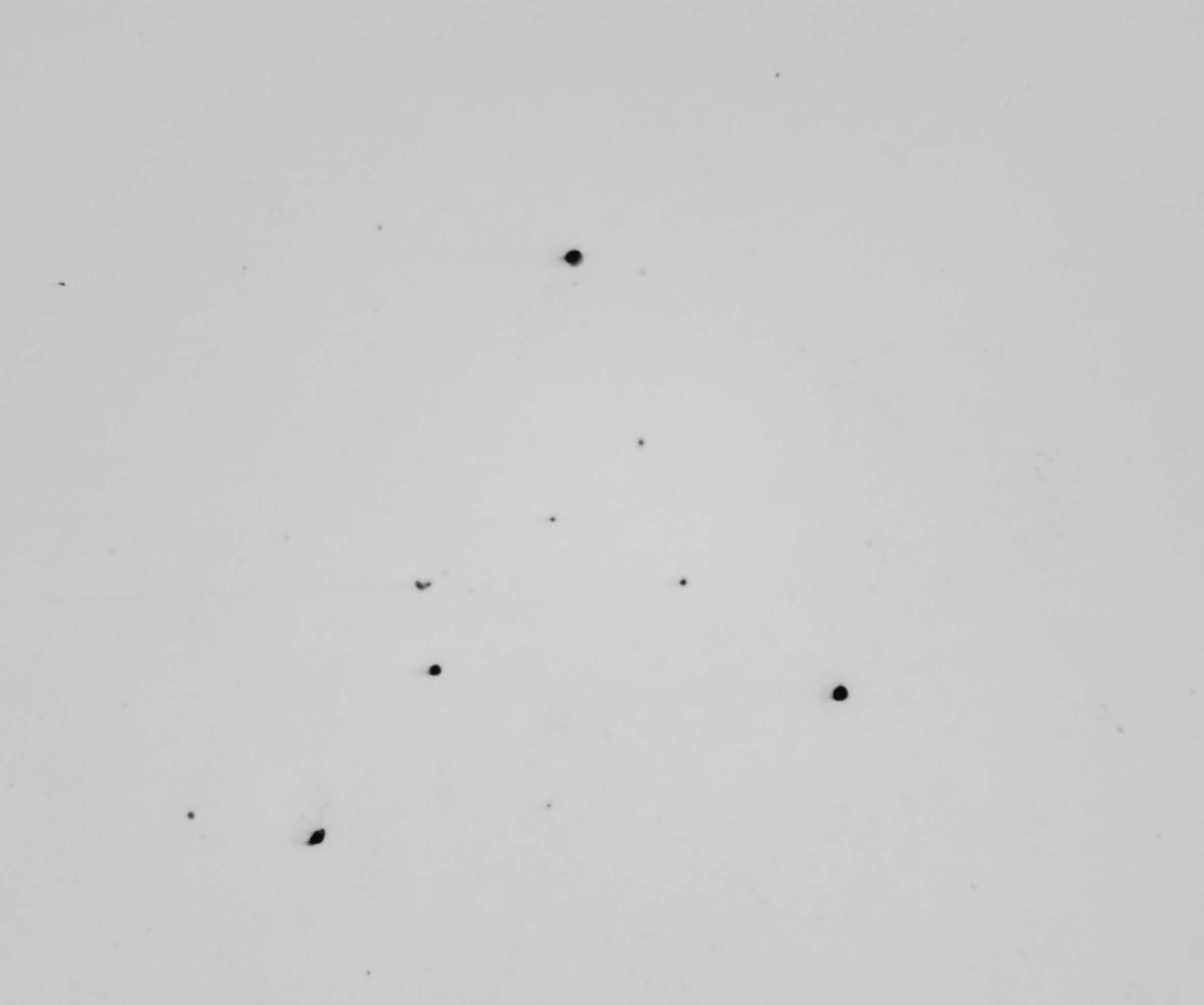

Supplement: Supplementary file 1 [file materials-17-01461-s001.zip › B3_2.jpg]

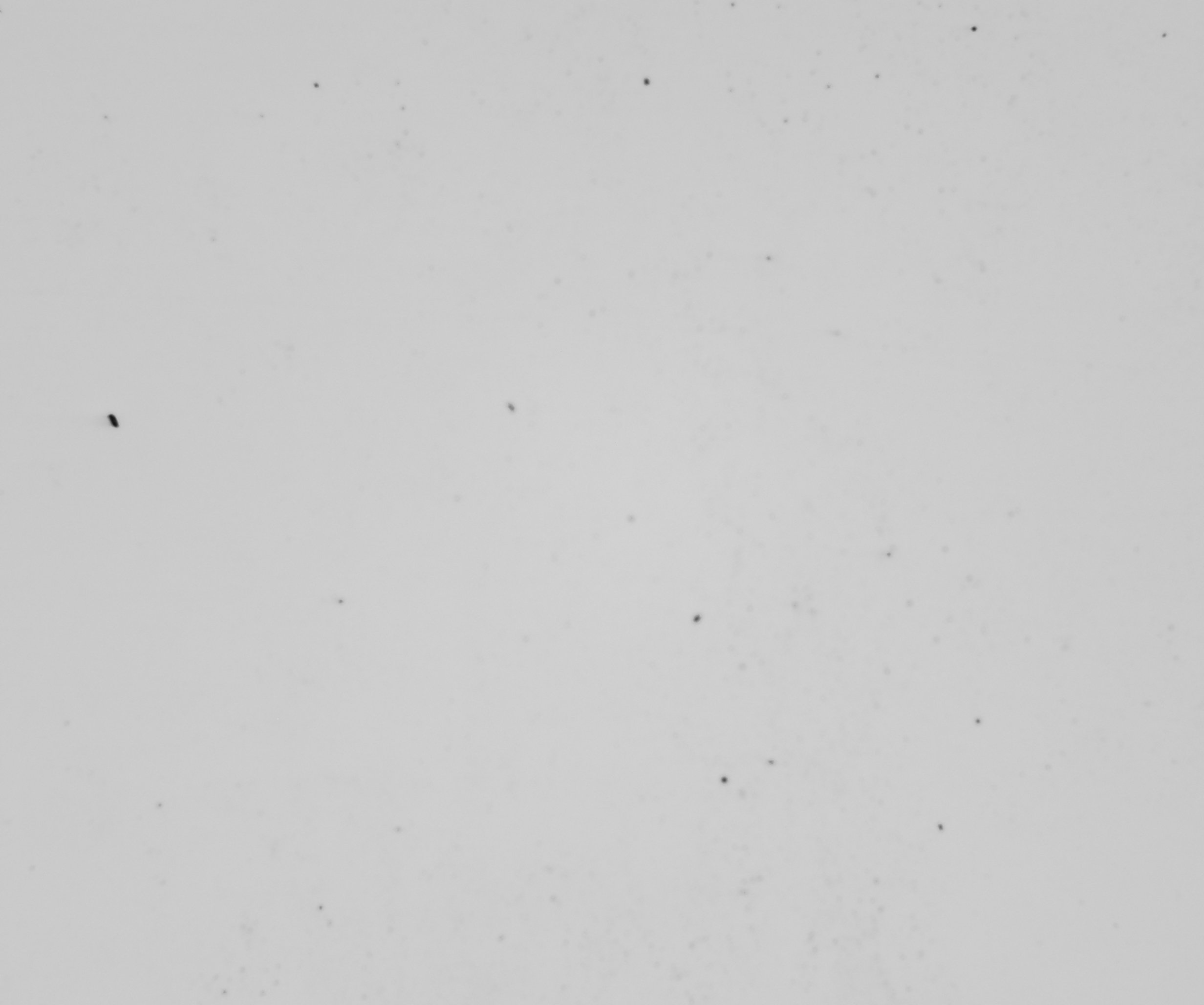

Supplement: Supplementary file 1 [file materials-17-01461-s001.zip › B3_3.jpg]

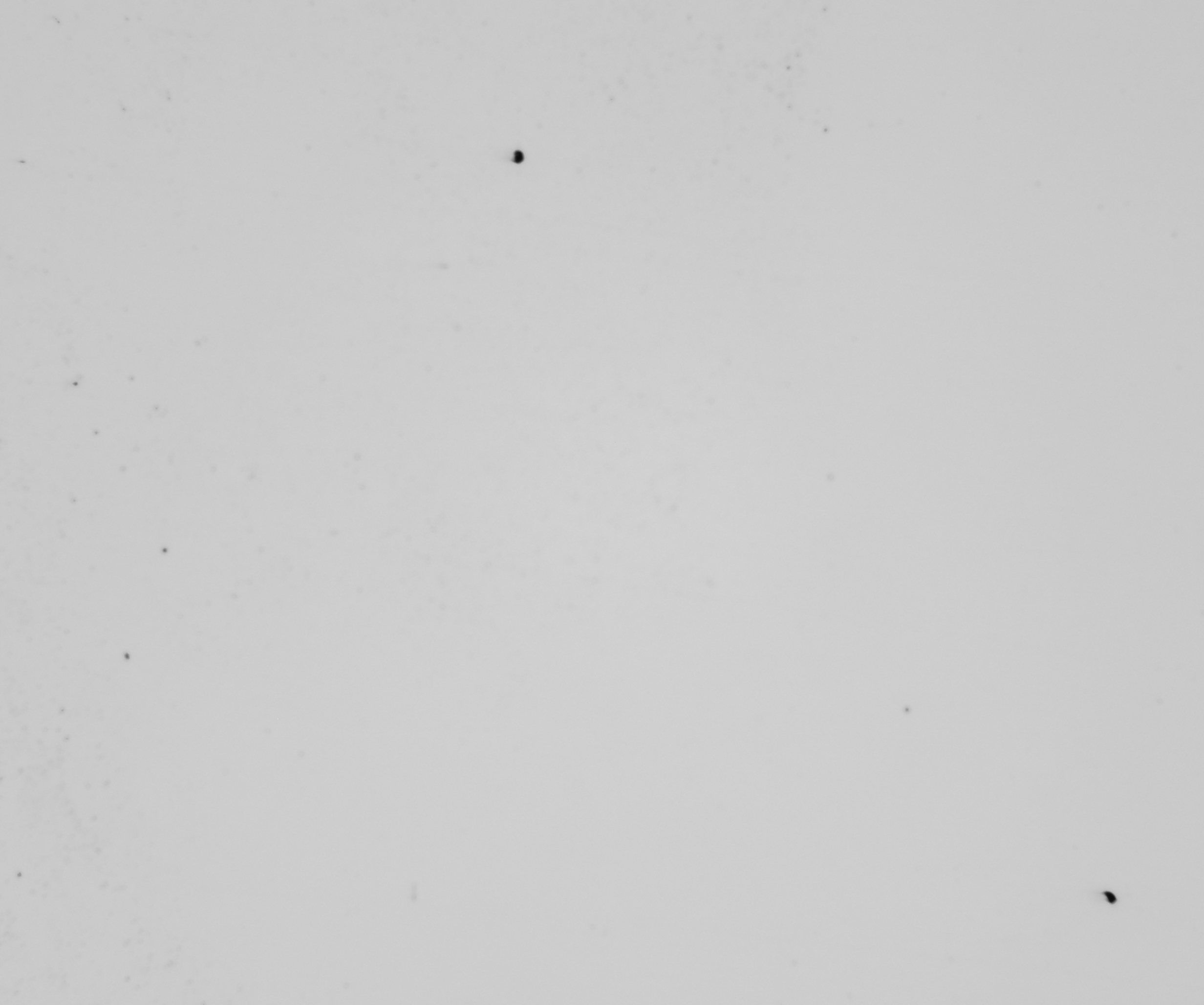

Supplement: Supplementary file 1 [file materials-17-01461-s001.zip › B3_4.jpg]

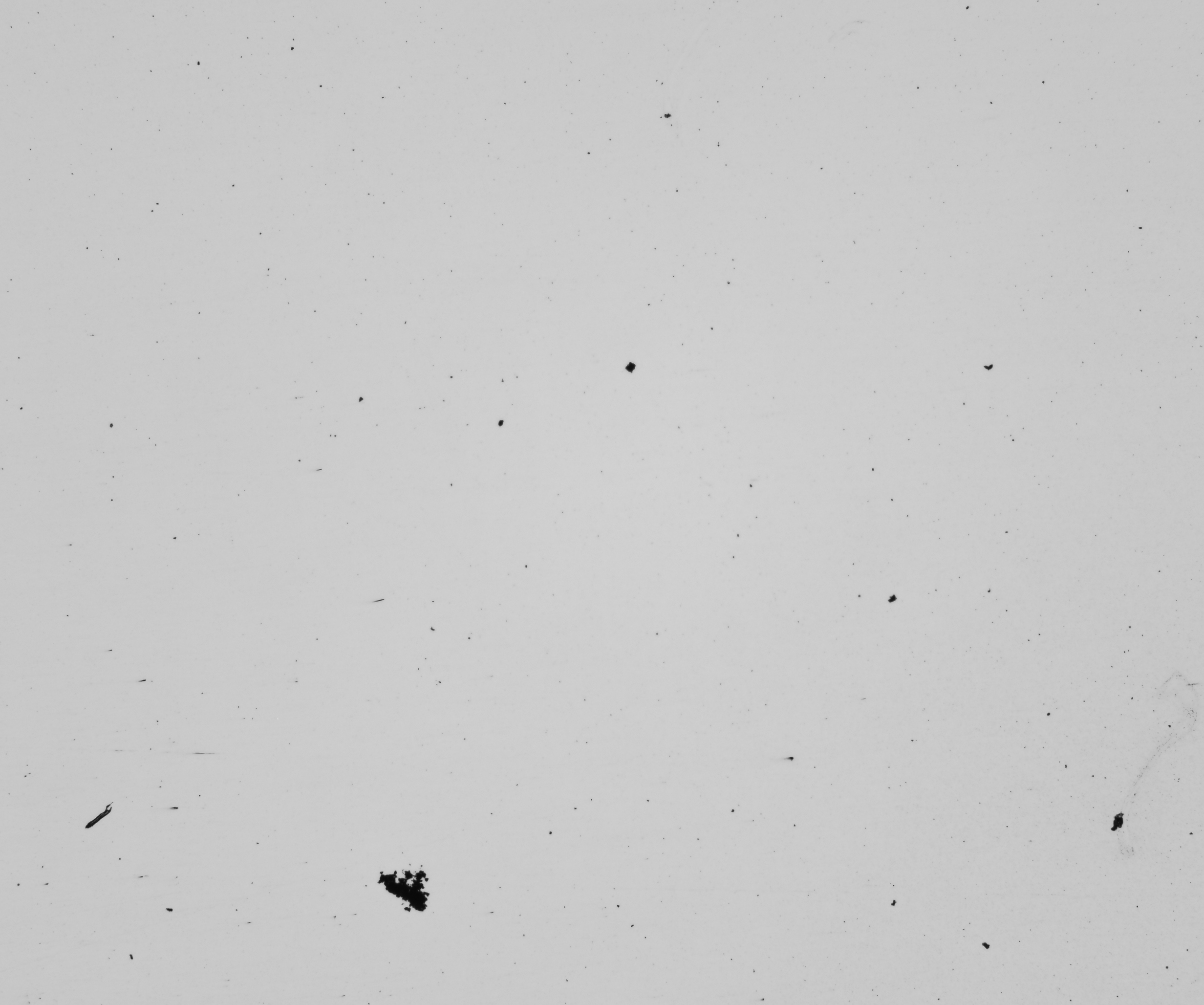

Supplement: Supplementary file 1 [file materials-17-01461-s001.zip › C1_2.jpg]

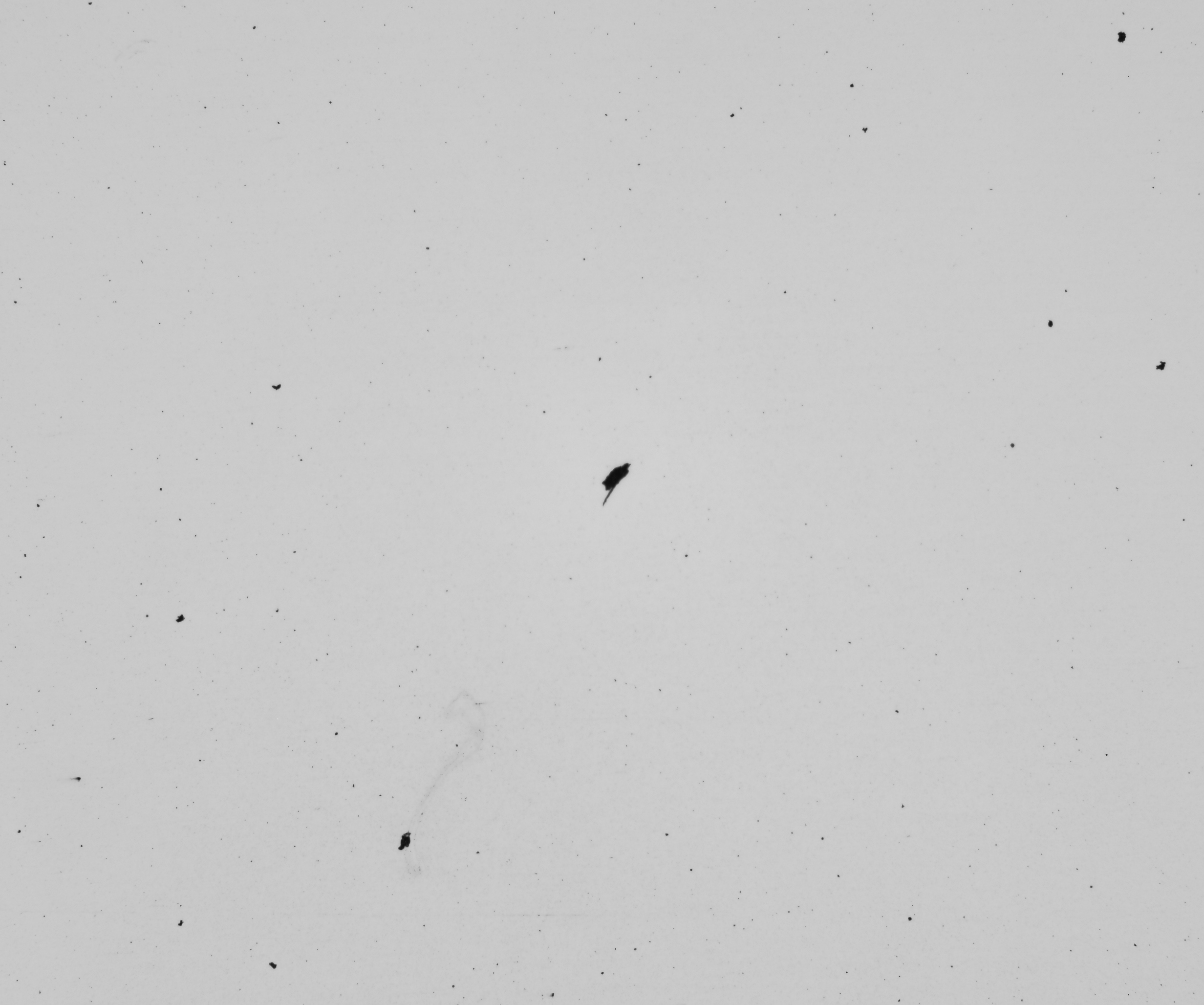

Supplement: Supplementary file 1 [file materials-17-01461-s001.zip › C1_3.jpg]

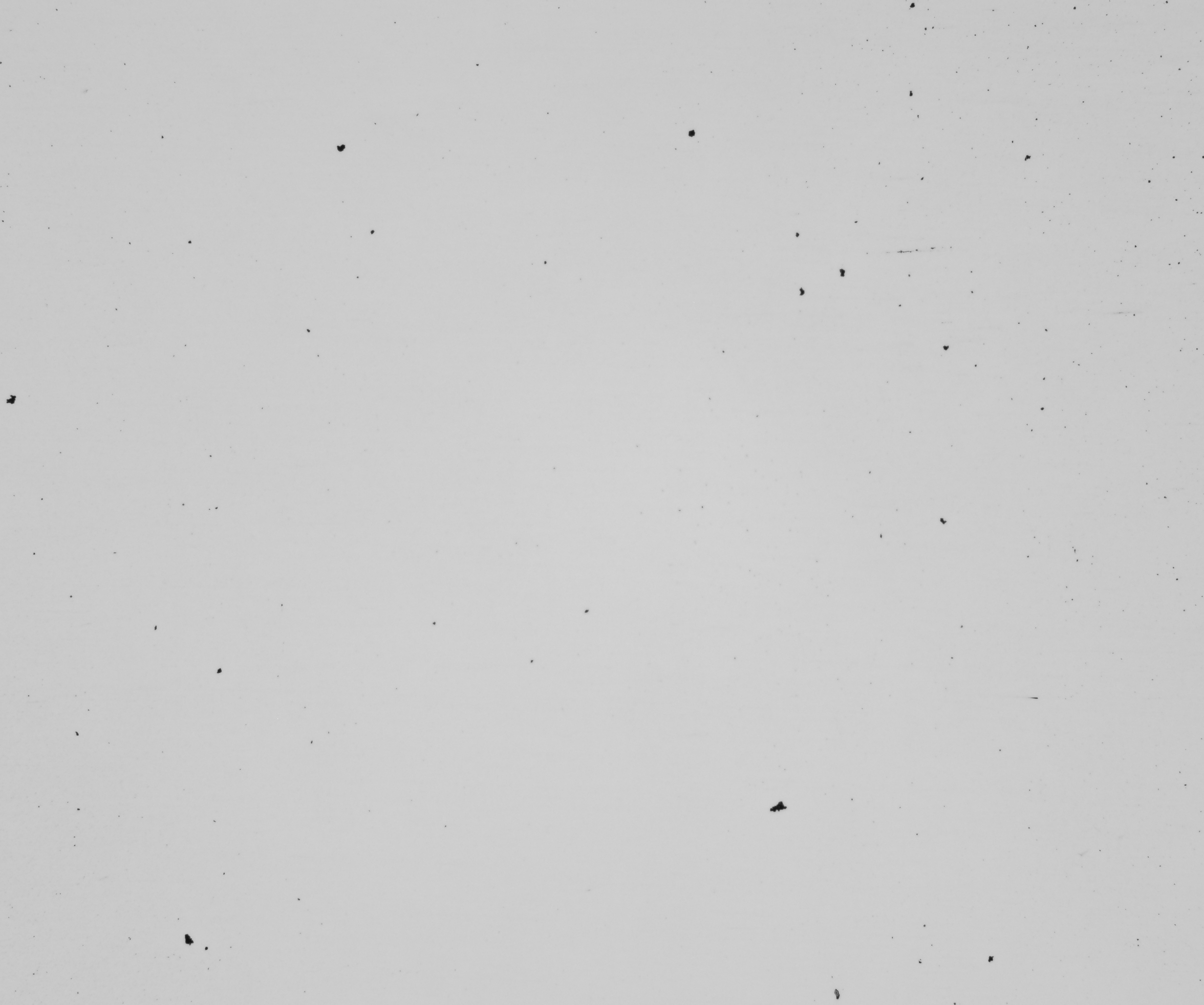

Supplement: Supplementary file 1 [file materials-17-01461-s001.zip › C1_4.jpg]

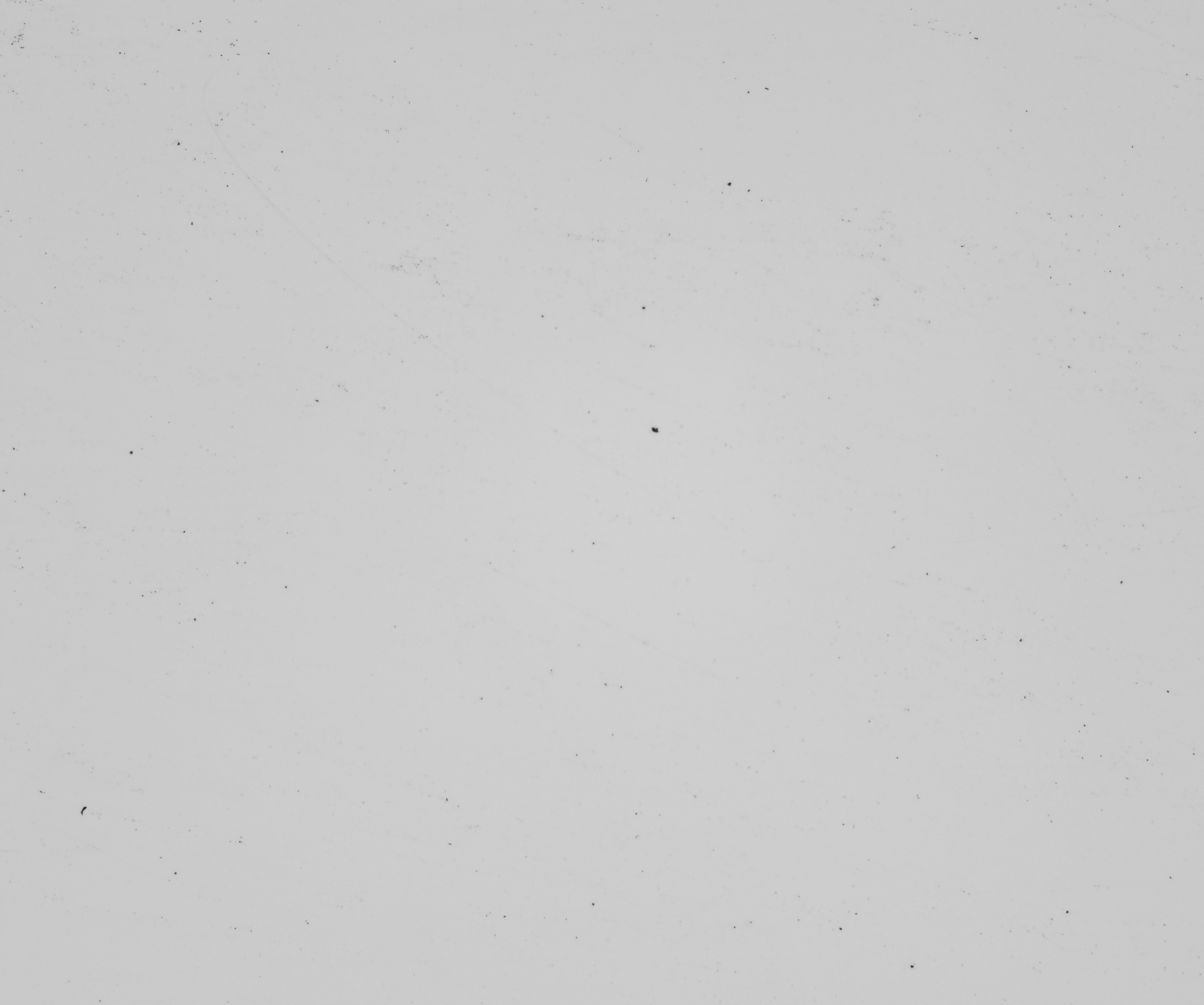

Supplement: Supplementary file 1 [file materials-17-01461-s001.zip › C2_2.jpg]

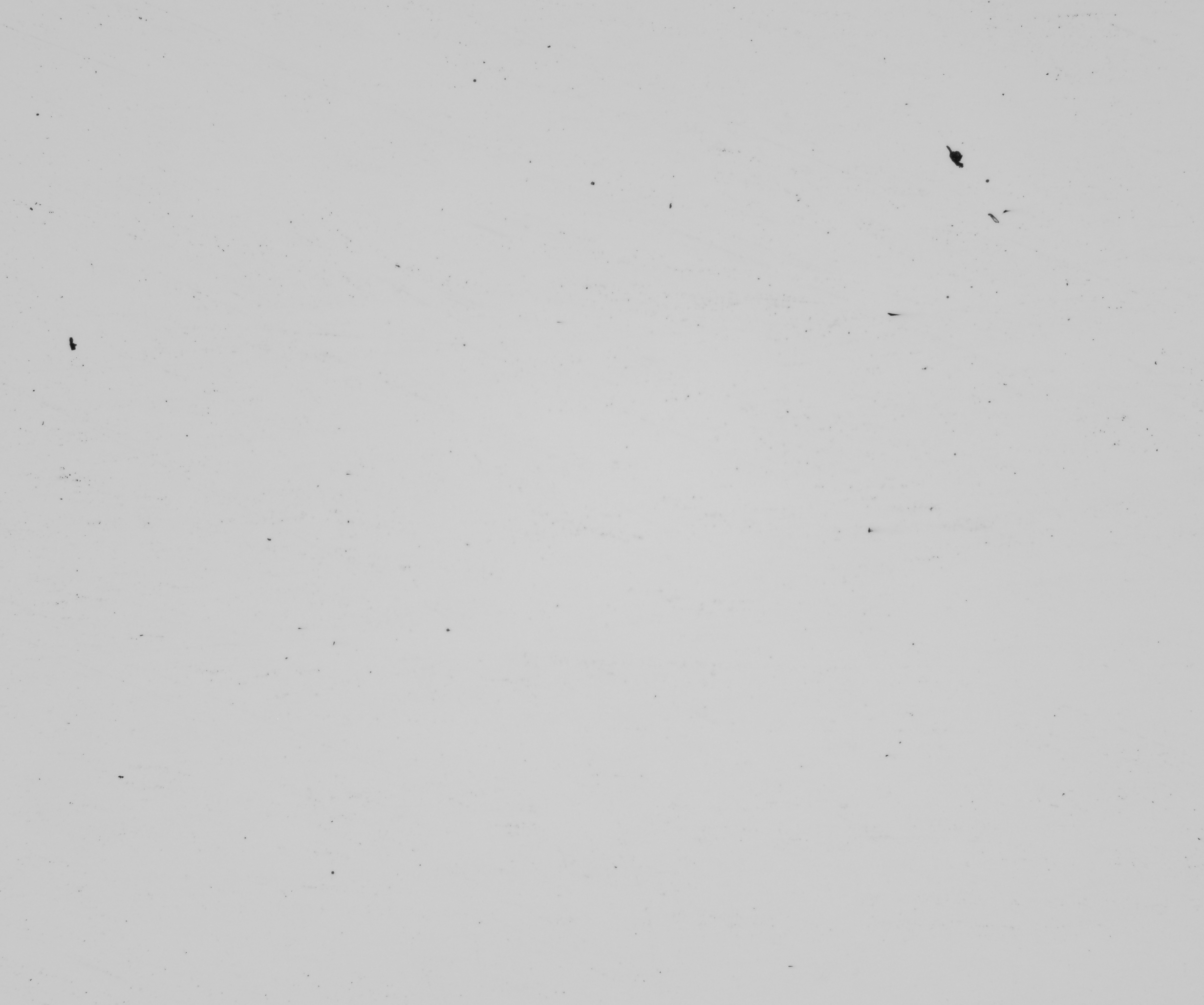

Supplement: Supplementary file 1 [file materials-17-01461-s001.zip › C2_3.jpg]

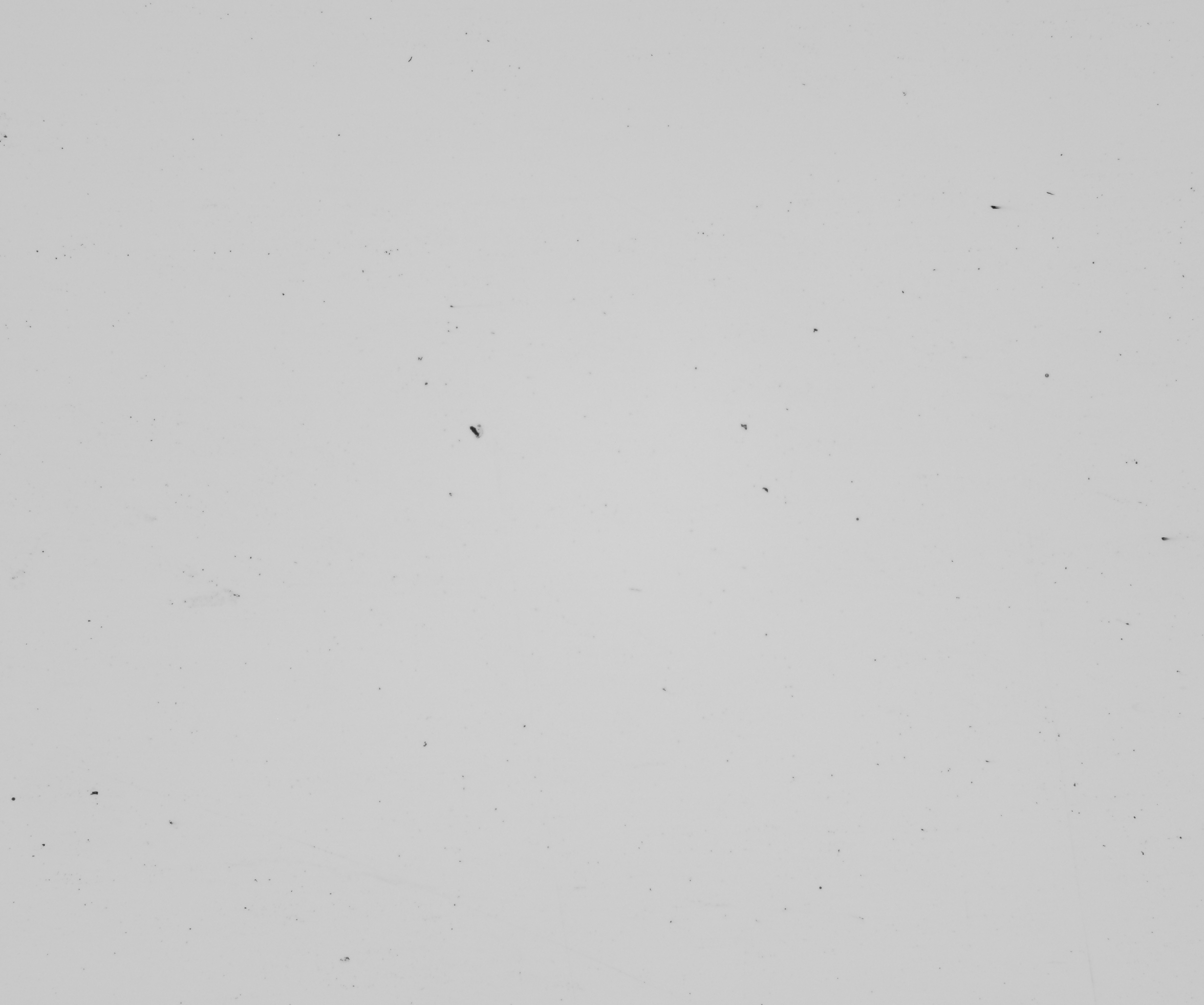

Supplement: Supplementary file 1 [file materials-17-01461-s001.zip › C2_4.jpg]

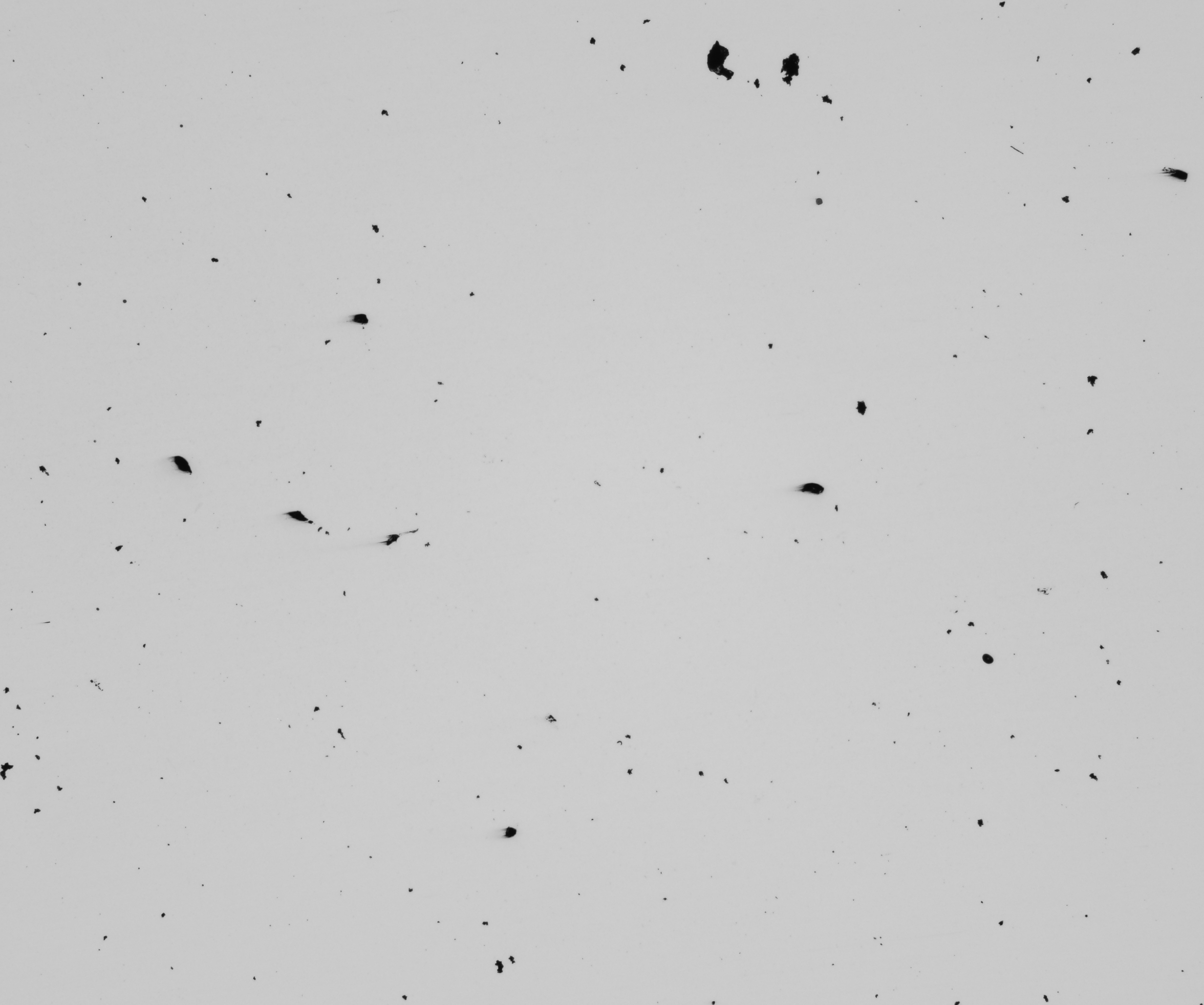

Supplement: Supplementary file 1 [file materials-17-01461-s001.zip › D1_2.jpg]

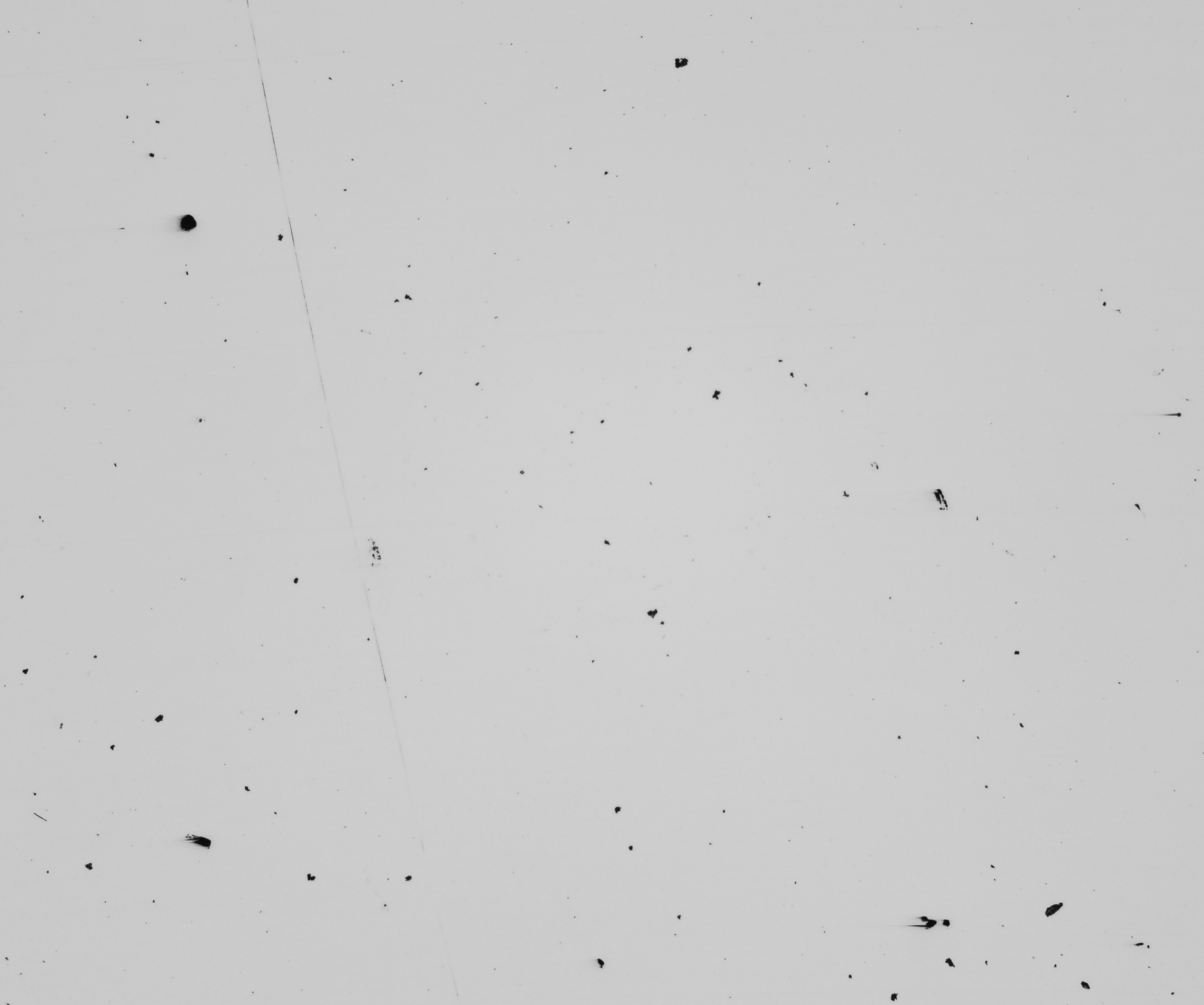

Supplement: Supplementary file 1 [file materials-17-01461-s001.zip › D1_3.jpg]

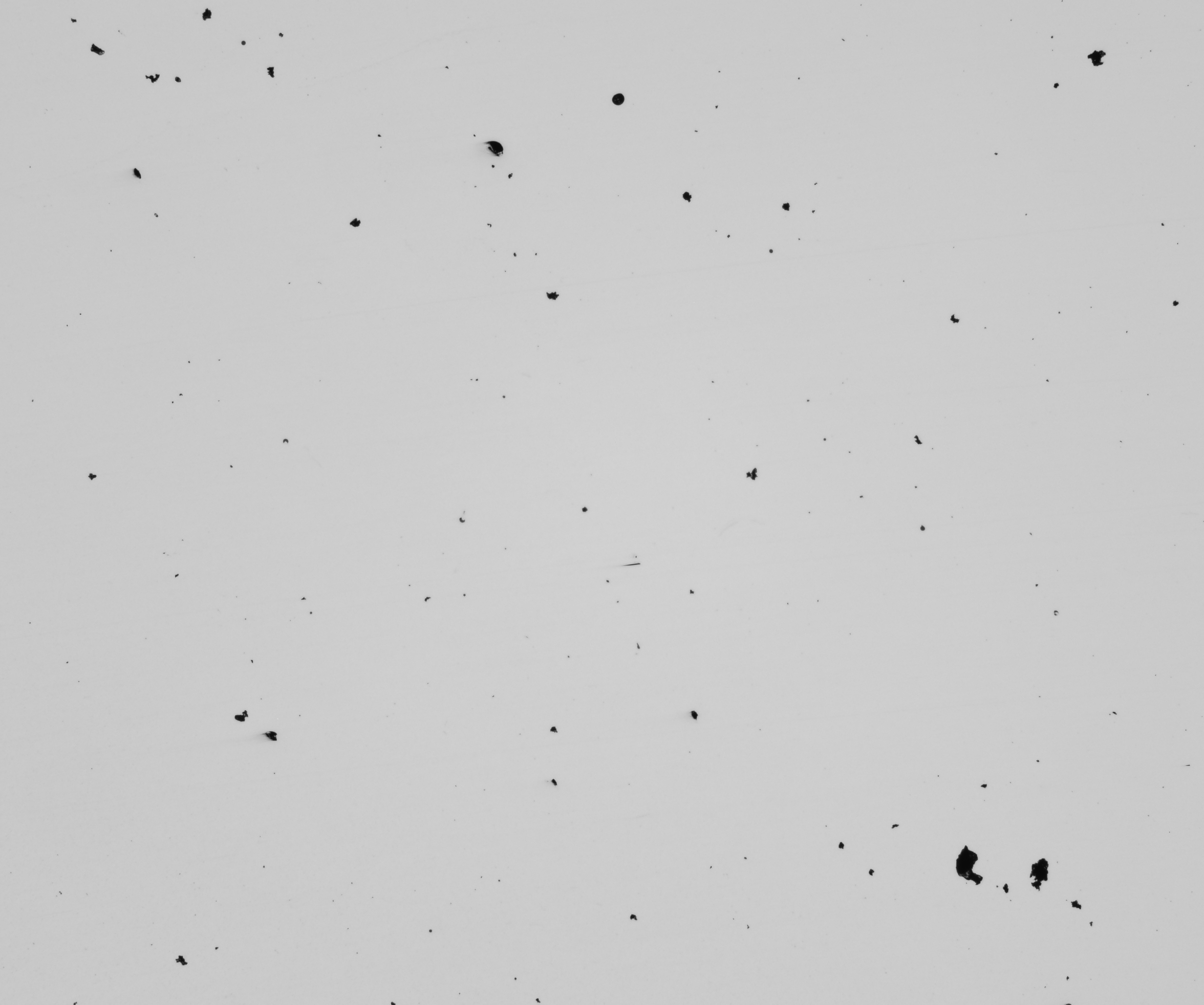

Supplement: Supplementary file 1 [file materials-17-01461-s001.zip › D1_4.jpg]

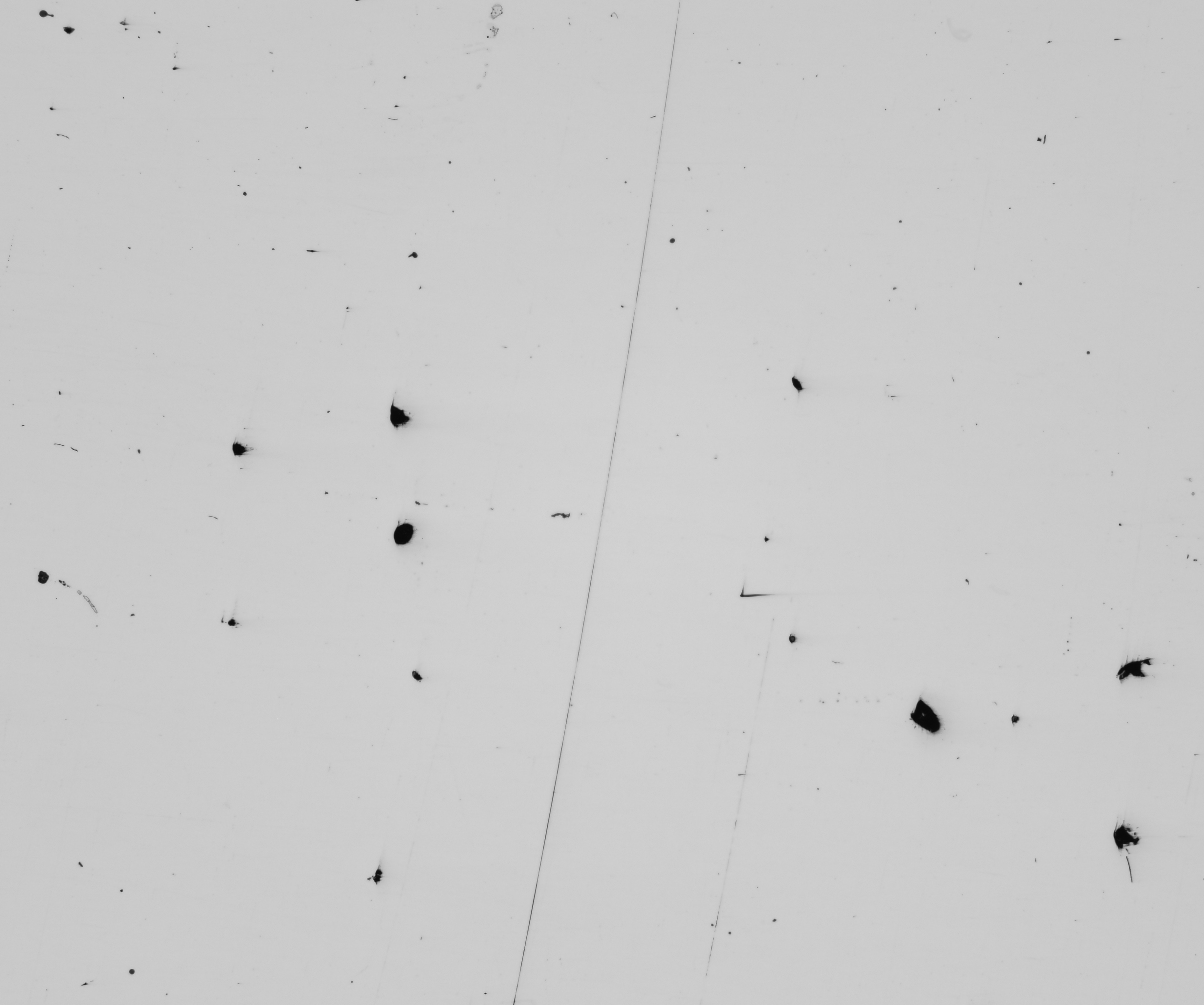

Supplement: Supplementary file 1 [file materials-17-01461-s001.zip › E1_2.jpg]

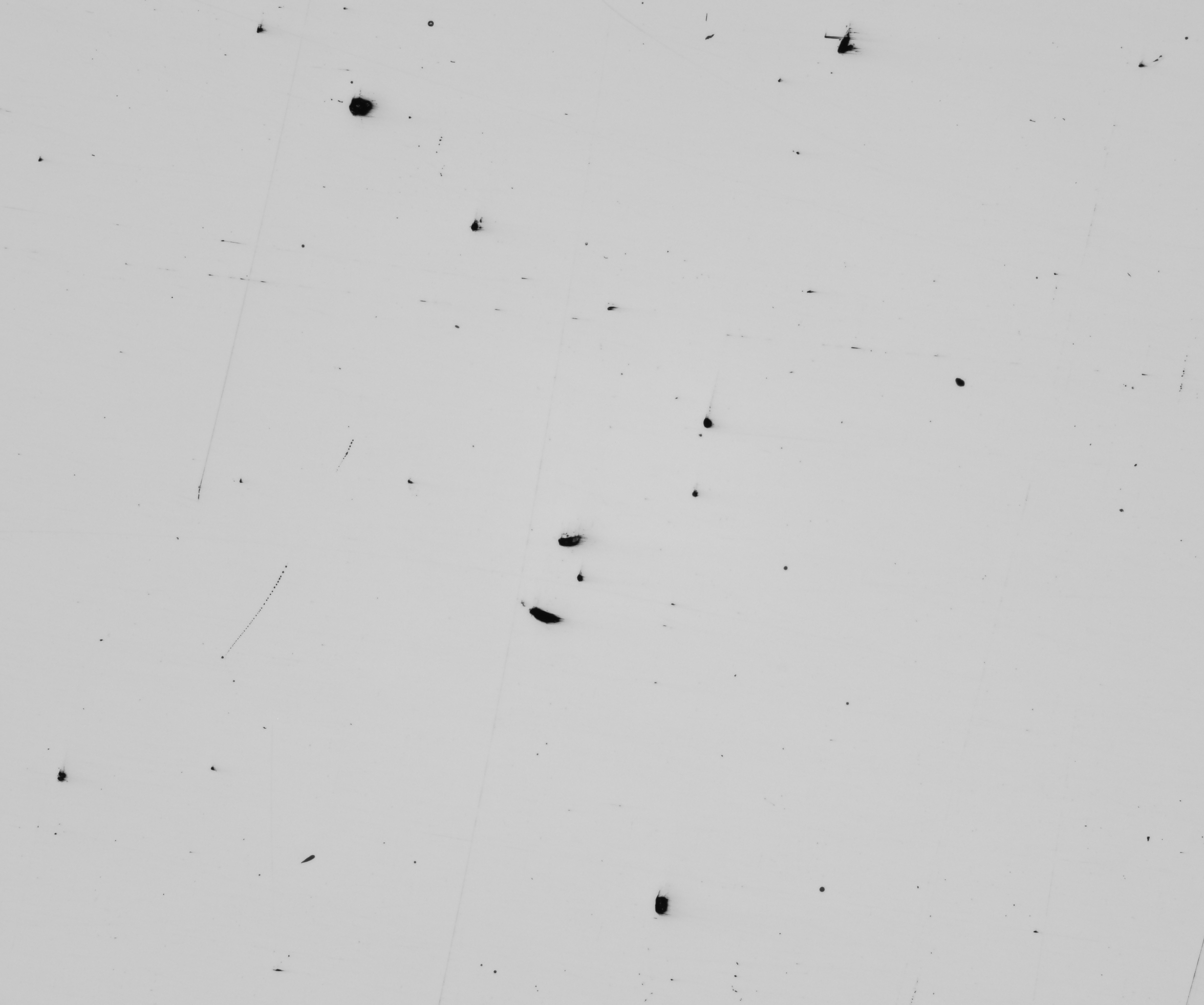

Supplement: Supplementary file 1 [file materials-17-01461-s001.zip › E1_3.jpg]

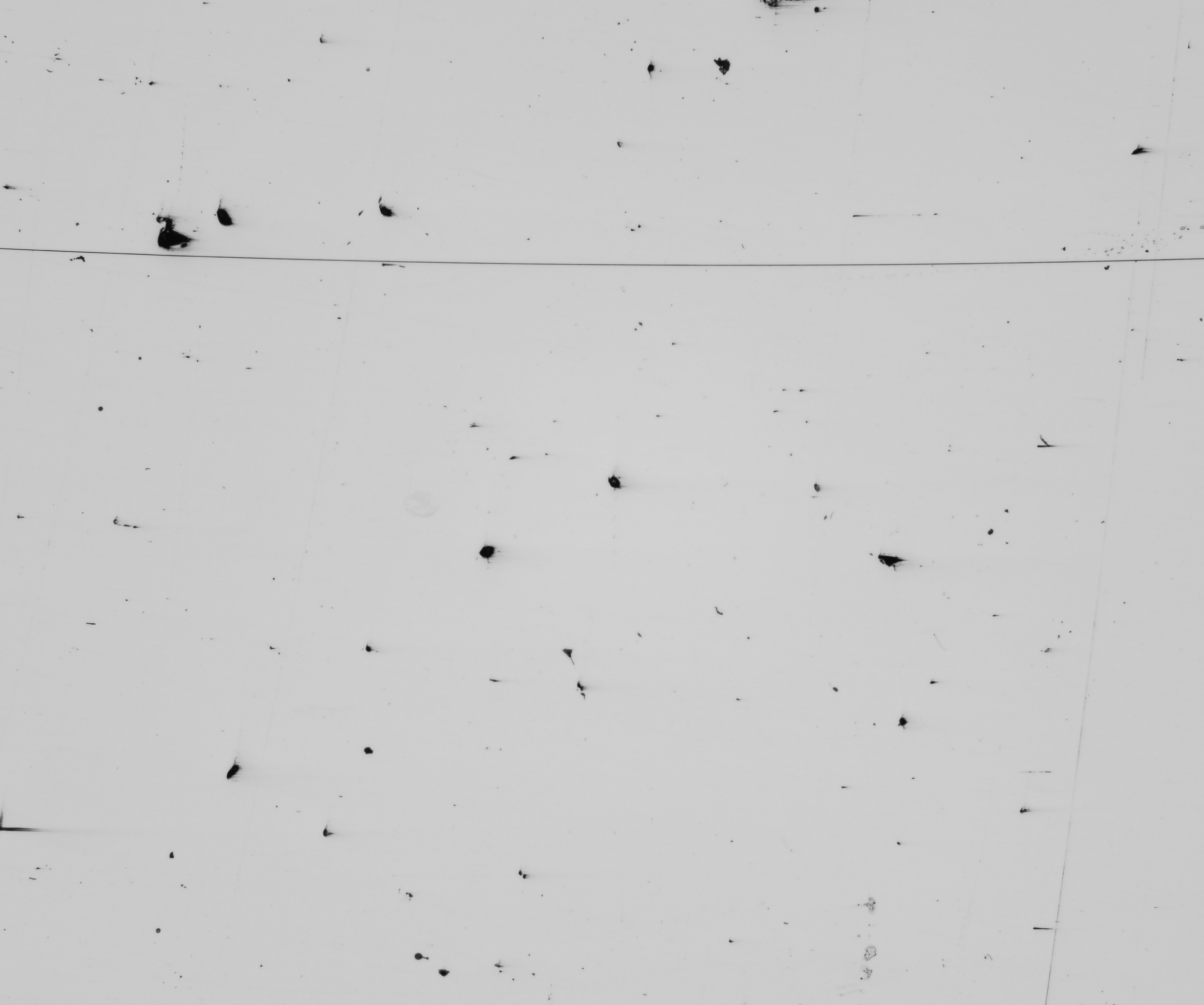

Supplement: Supplementary file 1 [file materials-17-01461-s001.zip › E1_4.jpg]
